# Mental health and socio-cognitive predictors of adherence to COVID-19 social distancing rules in adolescents in England

**DOI:** 10.1016/j.heliyon.2024.e41403

**Published:** 2024-12-20

**Authors:** Giacomo Bignardi, Saz P. Ahmed, Marc Bennett, Darren Dunning, Kirsty Griffiths, Jovita T. Leung, Ashok Sakhardande, Blanca Piera Pi-Sunyer, Willem Kuyken, Tim Dalgleish, Sarah-Jayne Blakemore

**Affiliations:** a Institute of Cognitive Neuroscience, University College London, UK; b Department of Psychology, University of Cambridge, UK; c Medical Research Council Cognition and Brain Sciences Unit, University of Cambridge, UK; d Department of Psychiatry, University of Oxford, UK; e Health Research Methods Unit, University of Hertfordshire, UK; f Bangor University, UK; g Betsi Cadwaladr University Health Board, UK; h School of Psychology, University College Dublin, Dublin, Ireland

## Abstract

The COVID-19 pandemic prompted governments worldwide to introduce social distancing measures, including school closures and restrictions on in-person socialising. However, adherence to social distancing was challenging for many – particularly adolescents, for whom social interaction is crucial for development. The current study aimed to identify individual-level influences on adherence to social distancing in a longitudinal sample of adolescents aged 11–20 years in England, who took part in a randomised controlled trial. At baseline, 460 participants completed detailed pre-pandemic assessments, including mental health and well-being, altruism, delayed reward discounting, rejection sensitivity, prosociality and susceptibility to prosocial and anti-social influence. Of these, 205 participants reported their compliance with COVID-19 social distancing rules and attendance at social gatherings between June and August 2020. Bayesian ordinal regression models were used to predict adherence to social distancing from predictors, controlling for age at pandemic, gender, day of assessment, and intervention group. The results indicated that higher levels of prosociality, altruism and lower susceptibility to anti-social influence were associated with higher adherence to social distancing. Pre-pandemic levels of depression, anxiety, delayed reward discounting, rejection sensitivity, conduct problems and emotional awareness were not robustly related to the outcomes. These findings have implications for understanding how adolescents comply with public health guidelines, highlighting the role of social influence and peer norms.

## Licencing

For the purpose of open access, the author has applied a Creative Commons Attribution (CC BY) licence to any Author Accepted Manuscript version arising from this submission.

## 1 Introduction

Beginning in 2020, COVID-19 posed a major public health challenge, leading to millions of excess deaths globally and unprecedented disruption to social, educational and economic activity [[1], [2], [3]]. In response, many countries introduced social distancing regulations to reduce transmission. In England, schools moved to online teaching for most students, and the Health Protection Regulations legally prohibited the size and type of social gatherings [4]. At the time, it was unclear how the public would adhere to these unprecedented measures and how they would impact population well-being [5]. In the current study, we aimed to investigate the factors that predicted compliance with COVID-19 social distancing regulations in a longitudinal cohort of adolescents (aged 11–20 years) in England.

Social distancing measures might be particularly challenging and potentially harmful for adolescents. Adolescence, defined as 10–24 years, is a developmental stage marking the transition to independent adulthood and the development of personal identity [6]. Compared with children, adolescents typically spend less time with family and more time with peers, as well as showing heightened sensitivity to social exclusion [7,8]. Social distancing may limit the autonomy and peer interactions crucial for adolescent social development [7]. Furthermore, concerns have been raised regarding the mental health consequences of depriving adolescents of peer interactions – particularly as pre-pandemic trends suggested a rising prevalence of depression and anxiety in young people – and increased demand for strained mental health services, which increased during the pandemic [9,10]. Some studies reported lower compliance with social distancing in young people than adults during the COVID-19 pandemic [[11], [12], [13]]. This is perhaps not surprising given the motivation of adolescents to engage with, and be socially influenced by, peers, while public health messaging has been primarily produced and enforced by adults [14].

Adolescents are thought to be particularly susceptible to peer influence. Time spent with peers increases during adolescence, and heightened sensitivity to social rejection might partly motivate peer conformity [7,15]. Peers also influence adolescent health behaviours; for example, friends' tobacco and alcohol consumption predict adolescents' own use [16]. While adolescence is often characterised as a period of heightened risk-taking with peers [17], peers can also promote prosocial behaviours in adolescence, such as charitable giving [18]. If one's peers disregard social distancing rules, young people might conform to their friends to avoid social exclusion [14]. Indeed, one study found that adolescent concern about maintaining reputation with friends predicted reduced self-reported social adherence with social distancing [19]. Conversely, peers modelling social distancing behaviours could also promote adherence to the rules and guidelines.

Adolescence has also been characterised by changes in self-control, risk-taking and sensation-seeking [20]. While under-developed self-control is central to many theories of adolescent-typical behaviour, self-control encompasses many different but related processes [21]. One component that might be relevant for predicting adherence to social distancing is delayed reward discounting, which measures an individual's preference for a smaller, immediate reward, relative to a larger, delayed reward [22]. Previous research has found a rapid shift in preference between 13 and 16 years of age, from smaller, immediate rewards to larger, delayed rewards [23]. Preference for more immediate rewards has been associated with various risky behaviours, such as drug use and gambling in adults [24], though in adolescence, the evidence is mixed [25]. Greater reward discounting might be associated with less compliance with social distancing if immediate rewards, such as socialising in person, are preferred over possible future benefits (e.g., not catching COVID-19, reducing community disease spread and avoiding getting into trouble). Indeed, a previous study of adults in the UK found an association between greater reward discounting and reduced adherence to social distancing measures [26].

Since the health risk posed by infection was relatively low for most healthy adolescents [27], adherence to COVID-19 guidelines could be considered a social dilemma [28]. Specifically, adolescents had to balance their developmental need for social interaction, versus adopting health behaviours with a wider societal benefit. Compliance with COVID-19 guidelines can be regarded as a type of prosocial behaviour, defined as acts intended to benefit others [29]. Individual-level differences in prosociality and social factors can drive prosocial behaviour [30]. One paradigm for studying prosocial behaviour is the dictator game, where a participant is asked to divide a sum of money with a second player. The second player is powerless over this decision, so monetary donations are interpreted as evidence of prosocial behaviour. Previous research in adults and adolescents has demonstrated that dictator game-giving and self-report questionnaire measures of prosociality predict compliance with COVID-19 guidelines [[31], [32], [33]]. This suggests that the tendency to behave prosocially may be an important factor influencing adherence to social distancing.

Several longitudinal studies have identified associations between social distancing measures and increased population-level depression symptoms in young people, with mixed findings for other mental health symptoms [34,35]. Those struggling with mental health problems may break social distancing rules by seeking additional social support from friends. The majority of existing cross-sectional studies indicate a weak correlation between increased depression symptoms during the pandemic and decreased compliance in adults [12,[36], [37], [38], [39]]. Research on adolescents is more limited but indicates a similar trend [38]. In an ecological momentary assessment study conducted in the Netherlands during the COVID-19 outbreak, university students reported their mood and behaviour multiple times daily. Dips in mood were associated with subsequent engagement in pleasurable activities, possibly to improve and regulate mood [40]. If low mood is associated with seeking in-person social support, it might lead to reduced social distancing. However, depression is also linked with a loss of energy and social withdrawal [41], which could lead to increased social distancing behaviours. A limitation of the cross-sectional literature is that concern about COVID-19 may confound associations between mental health variables and compliance with guidelines. We sought to investigate whether pre-pandemic mental health measures influenced health behaviours – specifically, social distancing – during the pandemic.

### 1.1 The current study

The COVID-19 pandemic underscored the role of behavioural policies, such as social distancing regulations, in mitigating the spread of emerging infectious diseases [42]. Understanding the factors that increase adherence to these measures is important for enhancing future readiness against novel infectious diseases, particularly when treatments and vaccines are unavailable.

Our primary goal was to explore how social decision-making, behavioural and well-being factors, measured pre-pandemic, related to later self-reported compliance with the UK's 2020 COVID-19 social distancing regulations [4]. We used data from a school-based mindfulness study [43], which included two time points of rich pre-pandemic baseline data from 460 adolescents aged 11–17, who were additionally tested during the COVID-19 pandemic in June–August 2020.

We tested how 11 pre-pandemic factors might relate to self-reported social distancing. We hypothesised that greater adherence to social distancing would be associated with higher prosociality, altruism, susceptibility to prosocial influence and anxiety scores. We hypothesised that lower adherence to social distancing would be associated with greater susceptibility to anti-social influence, depression symptoms, conduct problems, rejection sensitivity (Cyberball social exclusion task; Williams & Jarvis [44]) and delayed reward discounting [22]. We had no directional hypotheses regarding the association between emotional awareness or executive functioning and adherence.

## 2 Methods

### 2.1 Sample

460 11–16-year-olds from 12 secondary schools in London and Cambridgeshire were recruited to participate in the MYRIAD study. This project aimed to test the effectiveness of school-based mindfulness training on mental health [45]. More detailed information regarding the sample can be found in Dunning et al. [43]. The MYRIAD project also includes a much larger trial [46] with different participants. Results from the larger MYRIAD trial and the smaller study showed that the intervention had a minimal impact on mental health or cognitive test scores [43,46].

Participants took part in four assessment time points, with three occurring before the COVID-19 pandemic (see Table 1 for dates). Time 1 assessments occurred before the intervention. Participants were then randomly allocated to intervention (mindfulness training) or active control (student success skills course) groups. Time 2 assessments took place after completing the intervention, on average 75 days after Time 1. Because many of the same assessments were taken at Times 1 and 2, and due to a lack of an effect of the intervention, we averaged across these to obtain more reliable measures of our explanatory variables. An additional round of online data collection occurred after trial completion at Time 3. Time 3 data is not used here as only a limited range of assessments was administered. The final testing time point during COVID (Time 4) was conducted via an online survey between June 10th and August 7th^,^ 2020, on average 1061 days after Time 1 and 991 days after Time 2.

**Table 1:** Descriptive statistics.

	Time 1	Time 2	Time 4
N	460	404	210
Gender			
Male	154	146	73
Female	306	258	137
Age			
M	13.8	13.9	16.7
SD	1.35	1.28	1.40
Range	11.2–16.8	11.4–17.0	13.9–20.0
Testing Dates			
Start	Sep 2016	Nov 2016	June 2020
End	Dec 2018	Dec 2018	Aug 2020

*Note.* Data from Time 3 was not used in this paper. *N* refers to sample size at each time point, including participants that missed some assessments.

During the Time 4 data collection period, the UK government's COVID-19 advice and regulations were in flux (see Supplementary Materials for a timeline; [4,47]). Between June 1st and July 4th, England's Health Protection Regulations (Amendment 3) prohibited indoor social gatherings of more than two and outdoor gatherings of more than six, with some exceptions for work and education. Schools began a phased re-opening from June 1st^,^ 2020 for some year groups, though many did not return to school until September 2020. On July 4th^,^ 2020, England's second Lockdown law was enacted, which prohibited gatherings of more than 30 people. The second Lockdown law reflected a shift from banning social gatherings in law, to greater reliance on recommendations [47]. Throughout this period, official government advice recommended against gatherings of more than six and no more than two households. In August 2020, local Lockdown regulations remained in place in certain cities, though these did not include areas near the schools in the study.

### 2.2 Measures

#### 2.2.1 Outcomes

##### 2.2.1.1 Primary outcome: compliance with COVID-19 social distancing

Our primary outcome was self-rated compliance with guidelines. Participants were asked: “On average in the past month, to what degree have you been complying with the government advice on self-isolating/social distancing?”. Participants responded on a 5-point scale, from 1 (“Not at all”) to 5 (“Completely”). A higher score indicates greater compliance with social distancing rules and recommendations.

##### 2.2.1.2 Secondary outcome: attendance at social gatherings

Our secondary outcome was self-reported attendance at social gatherings, assessed with the question: “In the past week, how often have you gone to social gatherings (e.g., house parties, meeting up with friends)". Participants responded on a 5-point scale, from 1 (“More than once a day”) to 5 (“Rarely or never”). At the time of data collection, the rules around social gatherings were in flux, and social gatherings were permitted in some situations and under certain conditions. Therefore, this outcome does not directly assess compliance with rules or recommendations but instead might indicate an individual's caution concerning the spread of COVID-19. A higher score on this variable indicates lower attendance at social gatherings.

#### 2.2.2 Baseline predictors of social distancing

We tested 11 different predictors of social distancing. Five predictors were factor scores generated from a battery of questionnaires on well-being, behaviour and mental health: Anxiety, Depression, Executive Functioning, Conduct Disorder and Emotional Awareness. The remaining six predictors were altruism (dictator game), delayed reward discounting (monetary choice questionnaire), rejection sensitivity (Cyberball Task), and three measures from the social influence task: prosociality, susceptibility to prosocial influence and susceptibility to anti-social influence.

##### 2.2.2.1 Well-being, behaviour and mental health

Participants completed several mental health & well-being questionnaires at Time 1 & 2. These included the Revised Children's Anxiety and Depression Scale (RCADS), the Behavior Rating Inventory of Executive Function (BRIEF), the Strengths and Difficulties Questionnaire (SDQ), the Difficulties in Emotion Regulation Scale (DERS), the Warwick-Edinburgh Mental Well-being Scale (WEMWBS), the Center for Epidemiologic Studies Depression Scale (CES-D) and the Child and Adolescent Mindfulness Measures (see protocol). Including all subscales from these questionnaires yielded 30 validated measures of well-being and behaviour.

Questionnaire scales were summarised using sum scores. To account for infrequent missing responses, we took the average score on non-missing items for each participant and multiplied it by the number of non-missing items. This approach is equivalent to a sum scoring when no data is missing. If more than 20 % of the responses were missing for a participant, the sum score was coded as “missing".

Factor analysis was then used to reduce the 30 sum scores to 5 factor scores, described in *Statistical Analysis.*

##### 2.2.2.2 Altruism (dictator game)

In this version of the Dictator Game, participants were asked to split a hypothetical £5 payment with a charity selected from 4 options (NSPCC, Oxfam, Shelter, RSPCA). Responses were continuously scored from 0 (give 0 % of the money) to 1 (give 100 %), so a higher response indicates a more generous donation. At Time 1 & 2, the average donation was 64.0 % and 61.8 %, respectively (SD = 29.3 % & 32.7 %), and 27.3 % and 30.6 % of the sample said they would donate all the money. Responses at Time 1 and 2 were moderately consistent (r = .47, N = 284, 95 % CI [.37, .55]).

##### 2.2.2.3 Delayed reward discounting

The 27-item monetary choice questionnaire measures preferences for a smaller, immediate reward relative to a larger, delayed reward [48]. We calculated the average discount rate (K) across all magnitudes [22]. Higher scores indicate greater discounting of future rewards, i.e., a preference for smaller immediate rewards. As recommended, discount rates were log-transformed. Discount rates for small, medium and large rewards were z-scored and then averaged. The average discount rate was consistent across Time 1 and 2 (r = .68, N = 287, 95 % CI [.61, .74]).

##### 2.2.2.4 Rejection sensitivity (cyberball)

At Time 2, Cyberball 5.0 was used to measure rejection sensitivity (Williams & Jarvis. [44]; https://www.empirisoft.com/cyberball.aspx), which has been extensively used with adolescents (e.g., Sebastian et al. [49]; Will et al. [50]). The task requires participants to play a virtual ball-tossing game with two animated players. Participants are told that they are connected to other real players over the internet, but the programme controls these other players. The task begins in the inclusion condition, where one-third of the ball throws are randomly sent to the participant. Next, in the exclusion condition, after two throws to the participant, the “virtual” players exclude the participant from the rest of the game by not throwing the ball to them. Participants completed mood and anxiety questionnaires at three time points: baseline and after the inclusion and exclusion conditions.

Rejection sensitivity was assessed by measuring change in mood and anxiety from baseline following social exclusion. The mood questionnaire consisted of four items on a 7-point Likert scale, and the anxiety questionnaire consisted of six items on a 4-point Likert scale. Responses were sum scored (no missing data on individual items), and items were recoded so that higher scores indicate greater anxiety or lower mood. Mood and anxiety sum scores were similar after baseline (Mood: M = 2.85, SD = 1.07; Anxiety: M = 1.84, SD = .58) and inclusion conditions (Mood: M = 2.74, SD = 1.16; Anxiety: M = 1.80, SD = .57), but increased markedly after the exclusion criterion (Mood: M = 3.85, SD = 1.51; Anxiety: M = 2.16, SD = .67). Therefore, we averaged change in mood from baseline to exclusion and change in mood from inclusion to exclusion, and did the same for anxiety. These subsequent measures of change in mood and anxiety were also highly correlated (r = .74, N = 389); thus, we again averaged the two to generate a composite measure of rejection sensitivity. Difference scores were z-scored before averaging to account for differences in measurement scale between mood and anxiety.

##### 2.2.2.5 Prosociality and susceptibility to social influence

The social influence task employed a widely used paradigm developed by our research group [18,51,52]. In the task, participants are presented with 16 scenarios (randomly selected from 82 possible scenarios) and asked to indicate how likely they would be to engage in the action described by using a slider scale anchored at “Never” and “Always”. Scenarios were carefully selected to be age-appropriate and evoke a range of responses, with some being prosocial and some anti-social. After submitting an “initial rating”, participants are shown the (purported) average rating from 11 to 16-year-olds, called the “provided rating”. This provided rating was actually randomly generated in each trial, using a uniform distribution with 20 % and 80 % minimum and maximum points on the slider scale. Half of the scenarios described prosocial acts (e.g., “Give money to charity), and half described anti-social acts (e.g., “Make fun of a classmate”). Participants were then asked again how likely they would be to engage in the behaviour (rating 2). Responses were recoded so that a higher response indicated a more prosocial response across all scenarios and in both conditions (range: 0–1).

Three measures were estimated from the social influence task. First, prosociality was assessed by evaluating participants' initial ratings before viewing the provided rating. Higher responses indicate higher prosociality on the initial rating (i.e., more likely to engage in prosocial acts, or less likely to engage in anti-social acts). Second, we measured susceptibility to prosocial influence by estimating how the provided rating influenced the second rating when the provided rating was higher (more prosocial) than the initial rating. Second, we measured susceptibility to anti-social influence by estimating how much the provided rating influenced the second rating, when the provided rating was lower (less prosocial) than the initial rating. More details on the development of this task can be found in Ahmed et al. (2020).

#### 2.2.3 Covariates

Four covariates were included in the models: age at Time 4 (years); day of assessment (days from the start of Time 4 data collection); gender (dummy coded; 0 = female, 1 = male); and intervention group (dummy coded; 0 = control, 1 = intervention).

### 2.3 Statistical analysis

#### 2.3.1 Dimensionality reduction of well-being, behaviour and mental health variables

Factor analysis was used to reduce the 30 mental health sum scores at baseline to fewer interpretable factors. The minimum residual factoring method was used, and factors were rotated using an oblique method (oblimin) using the psych R package [53]. Parallel analysis and Velicer's minimum average partial test indicated that five factors were sufficient to describe covariation between mental health variables. We labelled these factors: Anxiety, Depression, Executive Functioning, Conduct Disorder and Emotional Awareness. The factor loadings of each variable onto each factor are visualised in the supplementary materials. Factors were labelled based on the indicator variables with the highest loadings. The factor loadings are presented in the supplementary materials.

#### 2.3.2 Social influence task

Participants completed the social influence task at both Times 1 and 2. We extracted three individual differences measures from the social influence task at each time point: overall prosociality, susceptibility to prosocial and susceptibility to anti-social influence.

***Prosociality***. We modelled individual differences in the average initial rating on the social influence task to estimate prosociality using a Bayesian mixed-effect regression model (see Supplementary Materials for more detail, including model results). The initial rating (presented prior to seeing the provided rating from “peers”) was used as the outcome variable. Both prosocial and anti-social scenarios were included in the analysis. Initial ratings were recoded so that a higher response represented more prosocial behaviour; that is, increased willingness to engage in prosocial actions or decreased willingness to act anti-socially.

Prosociality was estimated by including a random intercept for each participant. The random intercept captures how the expected initial response on the task differs across individuals, with higher participant intercepts indicating higher average prosocial responses. A random intercept for each scenario was also included. Because we observed heteroscedasticity in the outcome across individuals, we also allowed the variance of the error term to differ across individuals in the model. The model also accounts for the censored outcome variable, which has ceiling and floor effects at the maximum and minimum response on the slider. Estimates of prosociality at Time 1 were consistent with Time 2 responses (r = .68, 95 % CI [.62, .73], N = 359).

***Susceptibility to prosocial and anti-social influence***. We employed Bayesian mixed-effect regression models to measure pro- and anti-social influence susceptibility (see Supplementary Materials for more detail, including model results). The second rating is used as the outcome, again coded so that a higher response indicates more prosocial or less anti-social behaviour. The model estimates how much the provided rating influences each participant's second rating. The impact of prosocial and anti-social influence is estimated for each participant, via two parameters named delta^+^ and delta^−^ respectively. delta^+^ measures social influence when the provided rating is more prosocial (higher) than the initial rating, and delta^−^ measures social influence when the provided rating is more anti-social (lower) than the initial rating.

Data from all 16 trials for all participants is entered into the model (at Time 1, there are 7200 observations, from 450 participants). Each participant's initial rating on each trial was included as a fixed predictor. We created two variables named delta^+^ and delta^−^. First, we calculated delta, the difference between the provided and initial rating on each trial (delta = provided rating – initial rating). For delta^+^, all negative values of delta were set to 0. For delta^−^, all positive values of delta were set to 0. Therefore, delta^+^ signifies the positive differences between the provided and initial ratings. Conversely, delta^−^ represents the negative differences between the provided and the initial ratings. delta^+^ and delta^−^ were entered into the model as correlated random slopes for each participant. A global intercept and fixed effects for delta^+^ and delta^−^ were also included in the model.

#### 2.3.3 Modelling adherence to social distancing

We averaged scores on the pre-pandemic predictors across Time 1 and 2 (except rejection sensitivity, which was only available at Time 2). If only Time 1 or Time 2 data was available for a participant, the score from the available timepoint was used. Averaging was performed for two reasons. First, it avoids repeating the same analysis using data from Time 1 and 2 separately, which would increase the evidence threshold needed following multiple comparison corrections. Second, averaged scores should also be more reliable [54]. For transparency, analysis results using data from each time point separately without averaging are provided in the supplementary materials. All explanatory variables were z-scored before and after averaging, except for two binary covariates (intervention group and gender), which were dummy-coded. Additional steps for pre-processing data are outlined below.

Ordinal regression was used to model the adherence to social distancing outcome variables. Ordinal regression has two primary advantages. First, our outcome variables with five ordered categories are unlikely to satisfy the distributional assumptions of standard models [55,56]. Second, ordinal regression models do not assume that the gaps between consecutive response options (i.e., “Not at all”, “A little”, “Somewhat”, etc.) represent equal increases in the outcome being measured.

The brms package was used to fit Bayesian cumulative ordinal regression models with a probit link function [57]. For a more detailed description, see Bürkner & Vuorre [58]. This model assumes the observed ordinal variable (Y) emerges from an underlying normally distributed latent variable $\tilde{\left(Y\right)}$. Participants with a higher score on the latent variable are more likely to select a higher category (i.e., “Completely”) and less likely to choose a lower category (“Not at all”). Based on the data, the model estimates cut-points determining the relative probability of selecting each response category. The probit link function maps the unobserved latent variable $\tilde{\left(Y\right)}$ to the observed ordinal outcome response (Y) using the cut-points.

Our primary interest is in how the explanatory variables predict the outcomes, and helpfully, the cumulative ordinal regression model is relatively simple to interpret here. The latent outcome variable is regressed onto the explanatory variables ($\tilde{Y}=b_{1}x_{1}+b_{2}x_{2}+\ldots +\text{error}$). This aspect of the model is analogous to standard linear regression, except the outcome here is the unobserved latent variable $\tilde{Y}$, which follows a normal distribution with zero mean and unit variance. By z-scoring (centring and scaling) the predictor variables, we can interpret the ordinal regression coefficients similarly to standardised linear regression coefficients. They indicate how much the expected value of $\tilde{Y}$ changes for every standard deviation increase in the explanatory variable.

To estimate the relationship between each explanatory variable and the outcomes, we ran separate ordinal regression models for each of our 11 predictor variables, only controlling for the four covariates.

We opted against the standard approach of running a single model including all 11 predictors and 4 covariates. To understand causal relationships, the inclusion of covariates in a statistical model must be justified to avoid introducing additional bias [[59], [60], [61]]. For example, controlling for a mediating variable, or a variable measuring the same construct, can attenuate the effect of a predictor variable. Conversely, conditioning on a variable that is caused by both the predictor and outcome leads to *collider bias,* generating a spurious effect*.* We judged that the chosen covariates are unlikely caused by any of the 11 predictor variables. For example, intervention group membership is determined randomly. Including these four covariates may improve our estimates due to confounding if they are a common cause of the predictors and outcomes. Including covariates may also increase statistical precision if they cause the outcome, and thus reduce residual error [62]. Correlations between all variables are shown in Supplementary Fig. 3. Correlations between covariates and outcomes were mostly weak, except day of assessment, which predicted reduced adherence as the study progressed. Only age at Time 4 and gender correlated with some predictors.

#### 2.3.4 Missing data

Of the 210 participants participating in Time 4, 205 responded to the outcome questions regarding compliance with COVID-19 social distancing and attendance at social gatherings. Of these 205 participants, 18 had missing variables on a single pre-pandemic predictor, and none had missing data on covariates (see Supplementary Fig. 2). For each ordinal regression model, participants with missing data were excluded. The sample size of each regression model varied between 204 and 205 for all predictors except rejection sensitivity, which had a lower sample size of 191.

#### 2.3.5 Bayesian methods primer

The objective of Bayesian modelling is to estimate a probability distribution (the posterior distribution) that quantifies our beliefs in the value of a set of parameters. Using the posterior distribution, we can also estimate the expected value of each parameter (the point-estimates) and a credible interval. Credible intervals are roughly analogous to confidence intervals, but are defined as an interval with a specified (e.g., 95 %) probability of containing the true effect. Here, we report highest density intervals that contain the most likely parameter values. As with confidence intervals, when the credible interval is narrower, it indicates more certainty in the true value of an effect.

When interpreting our Bayesian ordinal regression coefficients, if the credible interval does not contain 0, this would indicate that the observed data is incompatible with a null effect [63]. As we are testing whether 11 explanatory variables are associated with our outcome, we use a more conservative 99.5 % credible interval (since $.995\approx 1-\frac{.05}{11}$) and test if these exclude 0 for our regression parameters.

## 3 Results

**Fig. 1:**
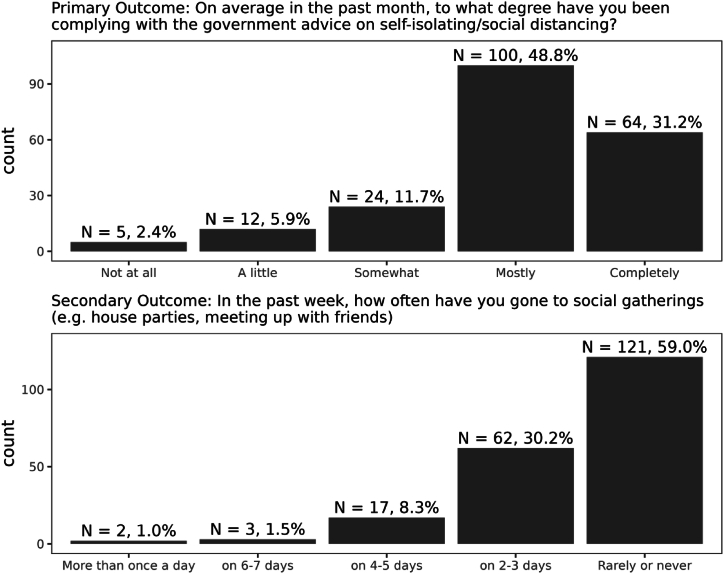
Response distribution of each self-reported social distancing outcome variable.

Descriptive statistics, including a correlation matrix of all variables, missing data, and response dates at Time 4 can be found in the Supplementary Materials. The response distribution for the primary and secondary outcome variables are shown in Fig. 1. Regression coefficients for each predictor can be found in Table 2.

**Table 2:** Standardised ordinal regression coefficients relating each explanatory variable to the primary and secondary outcomes, controlling for four covariates (age at Time 4, date of assessment, gender and intervention group).

Variable	Mean Est	95 % Credible Interval		99.5 % Credible Interval	
		LB	UB	LB	UB
**Primary Outcome (Social Distancing Compliance)**					
Anti-social Influence	−.271	−.455	−.087	−.531	−.007
Altruism (Dictator Game)	.270	.111	.427	.046	.497
Prosociality (Social Influence Task)	.262	.103	.423	.035	.493
Conduct Problems	−.184	−.353	−.013	−.427	.057
Depression	.127	−.040	.292	−.116	.368
Cyberball Rejection Sensitivity	−.125	−.293	.042	−.362	.112
Delayed Reward Discounting	−.039	−.232	.154	−.315	.241
Executive Functioning	−.033	−.193	.129	−.261	.198
Emotional Awareness	−.029	−.185	.129	−.251	.194
Prosocial Influence	−.010	−.182	.161	−.257	.238
Anxiety	.009	−.143	.165	−.209	.231
**Secondary Outcome (Attendance at Social Gatherings)**					
Altruism (Dictator Game)	.208	.039	.376	−.034	.454
Delayed Reward Discounting	.151	−.070	.374	−.165	.474
Prosocial Influence	.126	−.065	.323	−.150	.413
Conduct Problems	−.103	−.282	.079	−.359	.156
Emotional Awareness	.086	−.084	.257	−.160	.332
Anti-social Influence	−.065	−.258	.131	−.342	.215
Cyberball Rejection Sensitivity	−.032	−.208	.147	−.280	.222
Anxiety	−.019	−.183	.147	−.256	.217
Depression	.003	−.170	.176	−.244	.253
Prosociality (Social Influence Task)	.002	−.162	.169	−.230	.243
Executive Functioning	−.002	−.173	.168	−.246	.242

*Note*. LB and UB refer to the credible intervals' lower and upper bounds. The effects of the covariates on the outcome are shown in the supplementary materials.

### 3.1 Primary outcome: predicting social distancing compliance

We fitted ordinal regression models testing each explanatory variable separately while controlling for four covariates (age at time 4, day of assessment, gender and intervention group). Model convergence was good in all cases, with Rhat values less than 1.01 and effective sample sizes over 20,000 (see supplementary materials for more detail). Information on the covariate effects is reported in the supplemental materials; only the date of Time 4 assessment had a moderate negative effect (B ≈ −.17) across models, indicating reduced social distancing over time. After omitting cases with missing data, the sample size for each analysis was 205 for all models except those including dictator game altruism (N = 204) and cyberball rejection sensitivity (N = 191).

**Fig. 2:**
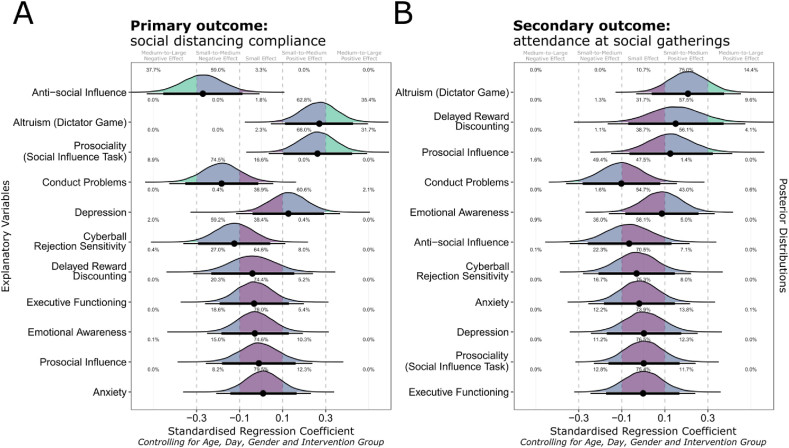
Ordinal regression coefficients for predicting social distancing compliance (Panel A) and attendance at social gatherings (Panel B). *Note*. Each panel shows the posterior probability distributions for ordinal regression coefficients relating each explanatory variable to the primary and secondary outcomes. Regression coefficients are adjusted for four covariates (age at Time 4, day of assessment, gender, and intervention group). Points indicate the distributions' median, representing each regression coefficient's central estimate. Thick and thin bars around each point indicate the 95 % and 99.5 % credible intervals, respectively. The posterior probability of each regression coefficient being small (between −.1 and .1), small-to-medium (between .1 and .3 or between −.1 and −.3), and medium-to-large (greater than .3 or less than −.3) are given in the plot. As described in *Method*, each effect is estimated from a separate regression model controlling for age at Time 4, day of assessment, gender and intervention group, and not from a single combined regression model with all 11 predictors in the same model.

Our results indicated that three factors were robustly associated with social distancing compliance. Adolescents more susceptible to anti-social influence were less likely to report complying with social distancing (B = −.271, 95 % CI [-.455, −.087], 99.5 % CI [-.531, −.007]). In contrast, prosociality measured by the initial rating in the social influence task (B = .262, 95 % CI [.103, .423], 99.5 % CI [.035, .493]), and altruism measured on the dictator game (B = .270, 95 % CI [.111, .427], 99.5 % CI [.046, .497]), were associated with greater compliance. The posterior probability distributions (see Fig. 2A) indicated that these effect sizes are probably medium-to-large and unlikely to be small (defined as less than .10 in magnitude).

The remaining predictors were more weakly associated with compliance. However, given the relatively small sample size in the present study, we lack the statistical precision to say that any of these other effects are definitely very small and ignorable. For example, we found a minimal association of anxiety with compliance (B = .009), however, there is a 12 % probability that this effect is greater than .10, and an 8 % probability that this effect is less than −.10.

We observed notable but uncertain trends that greater conduct problems (B = −.184, 95 % CI [−.353, −.013], 99.5 % CI [−.427, .057]) and greater rejection sensitivity (B = −.125, 95 % CI [−.293, −.042], 99.5 % CI [−.362, .112]) predicted slightly reduced compliance.

### 3.2 Secondary outcome: predicting attendance at social gatherings

Our secondary outcome was self-reported attendance at large social gatherings. This variable was coded so that a higher score indicated less frequent attendance (see Fig. 1). First, inspecting the posterior distribution of regression coefficients (Fig. 2B) suggests that most predictors had small but uncertain effects. Most 99.5 % credible intervals overlap with 0, indicating insufficient evidence for a non-zero effect.

Again, altruistic tendencies, as measured by the Dictator Game predicted fewer social gatherings (B = .208, 95 % CI [ .039, .376], 99.5 % CI [−.034, .454]). However, this effect was less certain, as we estimated a .86 % probability that the true effect of altruism is in the opposite, negative direction.

### 3.3 Sensitivity analysis

There are several reasonable approaches to model susceptibility to pro- and anti-social influence. Here, we fitted two alternative models and tested whether they had different associations with the primary compliance outcome. We kept the same model structure in all cases but changed the modelled response distribution. We used the ordered beta regression [64] and t-distribution robust regression models [60]. Our original estimate of susceptibility to anti-social influence at Time 1 correlated strongly with the estimate derived from the ordered beta regression model (r = .91, N = 450) and robust regression model (r = .67, N = 450).

The ordered beta-regression model estimate of susceptibility to anti-social influence remained a strong predictor of compliance (B = −.367, 95 % CI [-.569, −.164]). However, we found no relationship using a robust t-distribution regression model (B = −.015, 95 % CI [-.198, .168]). Moreover, the robust model suggested that between-subject variance in anti-social influence is extremely low, indicating that most individuals have similar levels of anti-social influence. Thus, there is some uncertainty in the association between susceptibility to anti-social influence and compliance, as this association depends on how social influence is modelled.

Most predictors were generated by averaging scores across two pre-pandemic testing time points. For transparency, we have presented the ordinal regression results using data from Time 1 and Time 2 separately without averaging in the supplementary results. The results are broadly similar, with anti-social influence, altruism, prosociality and conduct problems remaining the most significant predictors across analyses.

## 4 Discussion

Many effective public health policies rely on voluntary adherence from the public [65]. Therefore, understanding barriers and factors influencing compliance with public health guidelines is essential for preparedness for future pandemics. In response to the COVID-19 pandemic, many governments issued rules limiting physical gatherings and face-to-face contact. In England, these rules included school closures and limits on the number of social connections outside one's household. We explored which pre-pandemic psychological characteristics are associated with self-reported adherence to COVID-19 social distancing guidelines in a sample of adolescents in England. Our main finding was that higher prosociality and lower susceptibility to anti-social influence predicted increased compliance with social distancing recommendations. We found that the relationship between depression and anxiety on compliance is probably small. Our results highlight the role of individual differences in behavioural responses to public health recommendations.

Most adolescents reported complying “mostly” (100/205) or “completely” (64/205) with government advice on social distancing over the previous month. We identified several factors that predicted individual differences in adherence to social distancing guidelines. Higher prosociality (the initial rating on the social influence task) and altruism (dictator game) predicted greater self-reported adherence with social distancing. In contrast, greater susceptibility to anti-social influence predicted reduced adherence. Conduct problems, anxiety, executive functioning, delayed reward discounting, emotional awareness, depression, rejection sensitivity and susceptibility to prosocial influence were more weakly associated with compliance. None of our predictors was strongly related to the secondary outcome of self-reported attendance at social gatherings. However, we may have reduced precision to detect predictors of attendance at social gatherings, as most of the sample (59 %) responded that they “never” attended any. Furthermore, this outcome measure may be less sensitive as social gatherings were permitted in some forms during the time of data collection. In June 2020, outdoor gatherings of up to six people were permitted, and the legal restriction was then replaced in July with a “recommendation” against larger gatherings, and a legal restriction of gatherings of more than 30 [4].

Compliance with social distancing guidelines was associated with two baseline measures of prosociality. The first involved asking participants whether they would donate to charity. The second involved a social influence task which estimated participants' engagement in hypothetical prosocial and anti-social behaviours. Indeed, if the perceived risk of COVID-19 to one's own health is considered low (as it might have been for young people at the time of assessment [66]), it is logical that prosocial individuals are more likely to comply with health recommendations that primarily benefit others. This view is compatible with theories linking selfish behaviour in the dictator game to tendencies to maximise one's own utility above others [67]. Our results suggest that communicating the potential societal benefits of adherence to public health guidelines might be more effective than focusing on individual risk. Indeed, one study found that individuals who perceived the benefits of COVID-19 guidelines to be greater were also much more likely to report compliance (r = .64; [68]). It is also possible that effective communication strategies to promote compliance with public health guidelines differ depending on prosociality level. Future research could consider individual differences in prosociality in crafting targeted public health messages. Individuals with lower prosocial tendencies may respond to appeals to more immediate, personal benefits to adherence (e.g., reduced health risk).

This study also builds on the small evidence base linking dictator game giving to “real life” prosocial behaviour. One previous study has found that dictator game donations predicted whether participants would return a “misdirected” envelope of cash [69]. Our findings also support and extend an existing study that found that dictator game donations were associated with social distancing compliance in a sample of Swedish adults [31].

Peer influence is particularly salient in adolescence, motivated by the heightened perceived social risks of exclusion or loss of face [7,14,70]. Our study indicates that adolescents who were more susceptible to anti-social influence were less likely to adhere to social distancing guidelines, while susceptibility to prosocial influence had little association with adherence. To measure susceptibility to social influence, we tested how much participants' behaviour ratings were influenced by a provided “average” rating ostensibly from 11 to 16 year-olds, which was actually randomly generated by a computer program. We should note that most anti-social influence trials came from scenarios describing prosocial acts (e.g., “giving money to charity”), where the provided rating is more likely to be lower (less prosocial/more anti-social) than the participant's first rating. Thus, susceptibility to anti-social influence describes participants' susceptibility to disengage in prosocial acts. Susceptibility to social influence may only impact social behaviour when environmental opportunities are present. Therefore, there might have been more opportunities for anti-social influence from peers, such as invitations to attend large indoor parties, than for peers to encourage social distancing. This might explain why susceptibility to anti-social influence had a greater impact on adherence to social distancing guidelines than prosocial influence. The role of social influence highlights the need to involve adolescents in designing and implementing public health campaigns. Moreover, in future pandemics, it is crucial to consider the developmental needs of different age groups, like adolescents undergoing critical social and emotional developments, to adhere to strict social distancing guidelines.

Our research supports the existing literature on adults, highlighting the role of social influence in adherence to COVID-19 guidelines. Using an online international survey, Tunçgenç et al. [71], found that individuals’ perceptions of how closely their friends stuck to COVID-19 distancing rules were a strong predictor of self-adherence. Similarly, in the UK context, reports of prominent government figures breaking COVID-19 social distancing rules may have hastened the decline in population adherence [72].

Our sensitivity analyses showed that the choice of model to analyse the social influence task impacts the resulting estimates of susceptibility to social influence and its association with compliance. We compared two alternative models to the standard normal distribution regression model. The ordered beta regression model was used to analyse the task. This model is designed to model responses limited to an interval (i.e., between 0 and 1). We also used the t-distribution robust regression model, which can handle over-dispersion of the outcome variable (i.e., long tails). The normal and ordered beta regression model yielded similar estimates of social influence and associations with adherence. However, estimates of susceptibility to anti-social influence from the t-distribution model differed and were not associated with adherence to social distancing rules. Additional work is needed to identify best practices for modelling individual differences in susceptibility to social influence.

We did not find a clear effect of executive functioning on compliance with social distancing. Executive functioning was assessed using principal component analysis of the questionnaire measures, with the executive functioning component loading strongly (>.60) on the BRIEF measures of planning, working memory, task completion and organisation. Our results suggest the executive function likely has a small effect (74.4 % posterior probability), or a small-to-medium negative effect, indicating that greater executive function is associated with reduced compliance (20.3 % posterior probability). In contrast, working memory capacity, a key component of executive function [73], has been linked to greater compliance with COVID-19 in one online study by Xie et al. [74]. Another online study of undergraduate students also found an association between subjective executive functioning and social distancing behaviours [75]. The lack of a positive association between executive function and compliance in our study could be explained by several factors. First, behavioural ratings of executive function are related but do not map perfectly to performance-based measures [76]. Second, Xie et al. tested adults in the United States at the start of the pandemic in March 2020, when there were significant differences in social distancing guidelines and overall compliance and motivation relative to our UK-based sample.

Delayed reward discounting has been theoretically and empirically linked with the emergence of poor health behaviours such as alcohol and drug use [24,77]. However, we did not find conclusive evidence of an association between delayed reward discounting and social distancing adherence. Higher discounting rates have been conceptualised as poor self-control or an inability to delay gratification. Existing research has therefore focused on exploring links with health behaviours such as drug, alcohol and eating behaviours [78]. Such health behaviours might have more apparent deferred costs and benefits than social distancing behaviours. Suppose participants' perceived risks of non-adherence or the perceived benefits of adherence were low. In that case, reward discounting might be less relevant as participants would be less motivated to delay gratification for an ambiguous benefit.

Our study did not identify a strong relationship between well-being factors and adherence to social distancing, suggesting that the interplay between these variables may be more complex than previously assumed. We comprehensively reduced an extensive battery of 30 mental health questionnaires to five principal components, none of which strongly predicted adherence. Our analyses indicated an 80 % probability that the effect of anxiety is small (i.e., with a standardised effect less than .10 in magnitude). Elevated baseline depression symptoms were weakly associated with increased adherence to social distancing (B = .127, 95 % CI [-.040, .292]). The direction of this effect is not entirely certain, as we found an estimated 6.6 % probability that depression exerts the opposite effect. Notably, existing cross-sectional research in adults has found the opposite: that depression symptoms during lockdown predicted slightly decreased compliance (e.g., Guo et al., 2021; Koning et al., 2022; Solomou & Constantinidou, 2020; Xie et al., 2020). These studies have primarily relied on cross-sectional online mental health surveys conducted during COVID-19. However, a limitation of these designs is that we cannot separate the effects of COVID-19-related distress, such as fear of infection and risk perception [79], which may confound associations between well-being and social distancing.

Our findings speak against the idea, suggested at the start of the pandemic, that ‘mental fatigue’ and distress will significantly impact adherence to social distancing, leading to reduced adherence over time [80]. Of course, the short and long-term effects of social distancing measures on population well-being should be considered, but the existing literature suggests that mental health is a weak predictor of adherence to social distancing.

### 4.1 Limitations

First, as with many cohort studies conducted during the pandemic, the follow-up response rate was relatively low, reducing power and possibly leading to bias if responders differ markedly from the population [81]. However, a strength of the current study is that participants completed a detailed battery of assessments in person under controlled conditions over two pre-pandemic time points. We used average measures across these time points to increase measurement reliability and statistical precision [82]. Furthermore, although our sample size is smaller than some large online studies conducted during the pandemic, our participants were selected via schools as part of a randomised controlled trial, which should yield a more demographically representative sample with less risk of data integrity issues common in online surveys [83]. In addition, rather than simply relying on significance testing, which is limited in low-power settings, here we reported standardised effect estimates and posterior distributions to communicate uncertainty in our findings [84].

A second limitation is our reliance on self-reported measures of compliance. Social desirability bias could confound measures of pre-pandemic characteristics and compliance behaviour. Given that several studies have found that the predictors of COVID-19 health behaviours changed throughout the pandemic [13,33], a third limitation of our study is that we only have a single time point during the pandemic. Although we controlled for time in this study, responses were only surveyed in a short time window, months into the first UK lockdown. A fourth limitation is that time points 2–4 occurred following an intervention. However, we controlled for the intervention in the models and found no association with the outcome (see supplementary materials). Finally, we did not collect data on compliance from other household members, including parents. Research has found that parents’ rules and expectations [32] and their own adherence with social distancing [85] are moderately related to adolescent social distancing and adherence to lockdown rules. Parental monitoring and rules around social distancing may have constrained adolescents' behaviour, limiting the role of adolescent-level individual differences on their own adherence to guidelines.

While our study focused on the role of pre-pandemic individual differences, more intervention research is needed to understand how public health policies can effectively modify adolescent health behaviours. Our findings highlight the importance of considering developmental and individual differences factors when designing public health interventions for adolescents in the context of contagious disease transmission and other health behaviours.

## 5 Conclusions

This study aimed to explore the factors associated with adherence to COVID-19 social distancing regulations in a sample of adolescents in England. We found that higher prosociality and lower susceptibility to anti-social influence predicted increased compliance with social distancing recommendations. Our study highlights the potential for leveraging adolescents’ prosocial tendencies and peer influence in public health campaigns, such as involving young people in their design and production [14]. Given that adolescence is a crucial stage of development characterised by significant cognitive, emotional and social changes, the development of public health regulations should consider the impact on sensitive periods of development.

## CRediT authorship contribution statement

**Giacomo Bignardi:** Writing – review & editing, Writing – original draft, Formal analysis, Data curation. **Saz P. Ahmed:** Writing – review & editing, Project administration, Methodology, Data curation, Conceptualization. **Marc Bennett:** Writing – review & editing, Project administration, Conceptualization. **Darren Dunning:** Writing – review & editing, Project administration, Conceptualization. **Kirsty Griffiths:** Writing – review & editing, Project administration, Conceptualization. **Jovita T. Leung:** Writing – review & editing, Project administration, Conceptualization. **Ashok Sakhardande:** Writing – review & editing, Project administration. **Blanca Piera Pi-Sunyer:** Writing – review & editing, Writing – original draft, Project administration. **Willem Kuyken:** Writing – review & editing, Resources, Project administration, Investigation, Funding acquisition. **Tim Dalgleish:** Writing – review & editing, Writing – original draft, Project administration, Funding acquisition, Conceptualization. **Sarah-Jayne Blakemore:** Writing – review & editing, Writing – original draft, Supervision, Project administration, Funding acquisition, Conceptualization.

## Data availability statement

Data are available upon reasonable request. The data and codebook for the MYRIAD Trial are available from T.D. upon request (release of data is subject to an approved proposal and a signed data access agreement).

Analysis Code for the project can be found at https://osf.io/vbfnt/

## Ethical approval statement

This study was approved by University of Cambridge psychology research ethics committee (Reference: PRE.2015.067) and UCL psychology research ethics committee (Reference: 7199001). Informed consent was obtained from both participants and their parents prior to the study.

## Funding information

This study was funded by 10.13039/100010269Wellcome Trust, Grant/Award Number: WT107496/Z/15/Z. SJB received funding from the 10.13039/501100003986Jacobs Foundation; 10.13039/501100000265Medical Research Council; University of Cambridge; and the Wellspring Foundation.

## Declaration of competing interest

The authors declare that they have no known competing financial interests or personal relationships that could have appeared to influence the work reported in this paper.
