# Supplementary material for: Mental health and socio-cognitive predictors of adherence to COVID-19 social distancing rules in adolescents in England
Source: Heliyon. 2024 Dec 20;11(1):e41403. doi: 10.1016/j.heliyon.2024.e41403 (PMC11732665; doi:10.1016/j.heliyon.2024.e41403)
Supplement: Multimedia component 1 [file mmc1.docx]

# Supplementary Materials

## Additional Information: Prosociality Model

We used brms to fit a Bayesian censored mixed regression model (Bürkner, 2017). The output from this model is shown below (Supplementary Table 1 & 2). The BRMS output shows that the outcome variable is modelled using a censored normal (Gaussian) distribution, with an identity link for predicting the mean (mu) and a log-link for predicting the standard deviation (sigma) term. We will first focus on interpreting the parameters that influence the mean, then we will discuss those that influence the standard deviation term. The outcome (rating1_recoded) in a "long" format with each response on its own row, so that there are 7200 observations (450 participants each have 16 responses). The observed outcome has a standard deviation of .298 and a mean of .637. We used vague priors with a broad t distribution on each parameter, detailed in the table below. In this section we will refer to the results from the model estimated at Time 1.

The intercept for the mean is 0.66. As there are no other fixed effects, this indicates the expected response for the first rating from the model. We also included a random intercept for scenario (1 | scenario), which has a standard deviation of .13 (95% CI^[[1]](#footnote-2)^ [.11, .16]). Each random intercept for scenario accounts for how the average response differs across scenarios.

The most important parameter is the participant random intercept for predicting the mean response (mu Intercept). This random intercept captures differences in the expected prosociality rating for each participant. As shown in Supplementary Table 1, there is meaningful variability of these intercepts across individuals (SD(mu_Intercept) = .12, 95% CI [.11, .14]) relative to the standard deviation of the outcome variable (SD = .298). To get estimates of prosociality for each participant, we extracted the median of each participant’s random intercept across all MCMC draws.

To predict the variance of the ratings, we have both a global intercept term (sigma intercept) and a random intercept for each participant. Because the standard deviation term can only be positive, a log link is often used to predict this term from a set of predictors. The value of the global intercept term below is -1.20, but we exponentiate these values to get the variance of the outcome variable conditional on the variables in the model (Est = .298, 95% CI [.288, .308]). However, participants differ in the variability in their responses, as the random intercept term for participants was significant (Est = .35, 95% CI [.31, .38]). Note that this value is on the exponential scale.

## Supplementary Table 1

Results from Bayesian model estimating **prosociality** (initial ratings on the social influence task), at **Time 1**.

| **BRMS Output**  Family: gaussian  Links: mu = identity; sigma = log  Formula: rating1_recoded \| cens(censored_indicator_r1) ~ 1 + (1 \| scenario) + (1 \| id)  sigma ~ (1 \| id)  Data: dat_time1 (Number of observations: 7200)  Draws: 7 chains, each with iter = 4000; warmup = 1500; thin = 1; total post-warmup draws = 17500 | | | | | | | | |
| --- | --- | --- | --- | --- | --- | --- | --- | --- |
| **Variable** | **Estimate** | **Est. Error** | **95% CI LB** | **95% CI UB** | **prior** | **Rhat** | **Bulk ESS** | **Tail ESS** |
| **Group-Level (Random) Effects:** | | | | | | | | |
| **Participants (Number of Levels: 528)** | | | | | | | | |
| SD(mu Intercept) | .12 | .01 | .11 | .14 | student_t(7, 0, .75) | 1.00 | 5948 | 9288 |
| SD(sigma Intercept) | .35 | .02 | .31 | .38 | student_t(3, 0, 2.5) | 1.00 | 6505 | 10864 |
| **Scenario (Number of Levels: 82)** | | | | | | | | |
| SD(Intercept) | .13 | .01 | .11 | .16 | student_t(7, 0, .75) | 1.00 | 4212 | 7610 |
| **Population-Level (Fixed) Effects:** | | | | | | | | |
| mu_Intercept | .66 | .02 | .63 | .70 | student_t(3, .7, 2.5) | 1.00 | 2310 | 42217 |
| sigma_Interecpt | −1.20 | .02 | −1.24 | −1.17 | student_t(3, 0, 2.5) | 1.00 | 7747 | 10954 |

*Note*. For each parameter, Bulk_ESS and Tail_ESS are effective sample size measures, and Rhat is the potential scale reduction factor on split chains (at convergence, Rhat = 1).

## Supplementary Table 2

Results from Bayesian model estimating **prosociality** (initial ratings on the social influence task), at **Time 2.**

| **BRMS Output**  Family: gaussian  Links: mu = identity; sigma = log  Formula: rating1_recoded \| cens(censored_indicator_r1) ~ 1 + (1 \| scenario) + (1 \| id)  sigma ~ (1 \| id)  Data: dat_time2 (Number of observations: 5856)  Draws: 7 chains, each with iter = 4000; warmup = 1500; thin = 1; total post-warmup draws = 17500 | | | | | | | | |
| --- | --- | --- | --- | --- | --- | --- | --- | --- |
| **Variable** | **Estimate** | **Est. Error** | **95% CI LB** | **95% CI UB** | **prior** | **Rhat** | **Bulk ESS** | **Tail ESS** |
| **Group-Level (Random) Effects:** | | | | | | | | |
| **Participants (Number of Levels: 528)** | | | | | | | | |
| SD(mu Intercept) | .13 | .01 | .12 | .14 | student_t(7, 0, .75) | 1.00 | 5738 | 8970 |
| SD(sigma Intercept) | .34 | .02 | .31 | .38 | student_t(3, 0, 2.5) | 1.00 | 7059 | 10728 |
| **Scenario (Number of Levels: 82)** | | | | | | | | |
| SD(Intercept) | .13 | .01 | .11 | .15 | student_t(7, 0, .75) | 1.00 | 4574 | 7980 |
| **Population-Level (Fixed) Effects:** | | | | | | | | |
| mu_Intercept | .66 | .02 | .63 | .69 | student_t(3, .7, 2.5) | 1.00 | 2890 | 4861 |
| sigma_Interecpt | −1.24 | .02 | −1.28 | −1.20 | student_t(3, 0, 2.5) | 1.00 | 8998 | 12041 |

*Note*. For each parameter, Bulk_ESS and Tail_ESS are effective sample size measures, and Rhat is the potential scale reduction factor on split chains (at convergence, Rhat = 1).

## Additional Information: Susceptibility to prosocial and anti-social influence

We used brms to fit a Bayesian mixed regression model (Bürkner, 2017). The output from this model is shown below. The top line shows that the outcome variable is modelled using a normal (Gaussian) distribution, with an identity link for predicting the mean (mu). We will focus on the model fitted on Time 1 data, shown on Supplementary Table 3.

The outcome (rating2_recoded) is in a "long" format with each response on its own row, so that there are 7200 observations (450 participants each have 16 responses). The outcome has a standard deviation of .287 and a mean of .640. We used vague priors with a broad t distribution on each parameter, detailed in the Supplementary Table 3.

From the Population-Level fixed effects, we can see that the effect of prosocial influence (delta^+^ = .26, 95% CI [.22, .30]) is larger in magnitude than the effect of anti-social influence (delta^−^ = .12, 95% CI [.10, .14]). There is an approximately but not exact one-to-one relationship between the initial rating and second rating (B = .98, 95% CI [.96, .99]).

To find the expected value of rating 2 (for the average participant), we need to account for both the intercept (Est = .02), the main effect of rating 1 (Est = .98), and the effect of the provided rating through the two delta variables. Let us imagine a situation where the participant's initial rating is 0.50, and they are shown a "provided rating" of .60. This would correspond to a value of .10 for delta^+^ (.60 - .50) and 0 for delta^−^ . The effect of delta^+^ on the second rating is .26, and the effect of delta^−^ on the second rating is .12. The model would therefore predict that the second rating would be .536 (.02 + .50 × .98 + .10 × .26 + 0 × .12). Note that we allow the effect of delta^+^ and delta^−^ to differ between participants using random slope modelling, so the above only applies to the "average" participant.

The crucial part of the model for estimating individual differences in susceptibility to prosocial and anti-social influence are the random slopes for delta^+^ and delta^−^ . Every participant has their own slope for these two variables, capturing individual differences in how the provided rating impacts the second rating. Interestingly, we observe greater variability in the delta^+^ effect between participants (SD(delta^+^) = .25, 95% CI [.23, .28]) relative to the effect of delta^−^ (SD(delta^−^) = .12, 95% CI [.11, .14]). Participants who are more susceptible to anti-social influence were also more susceptible to prosocial influence (cor(delta^+^, delta−) = .39, 95% CI [.25, .51]). This correlation is moderate, indicating that these represent constructs that are reasonably distinct.

For each scenario, we also included random slopes that account for differences in the effect of delta^+^ and delta− across each scenario. Because 16 scenarios out of a possible 82 are randomly presented to each participant, these random slopes control for which particular scenarios were presented. Indeed, different scenarios are associated with different levels of pro- and anti-social infleunce. Interestingly, we observe slightly greater variability in the delta+ effect between scenarios (SD(delta+) = .09, 95% CI [.06, .12]) relative to delta^−^ (SD(delta^−^) = .05, 95% CI [.03, .06]).

To predict the variance of the second rating, we did not include a random effect for participant in this model, as this led to issues with model fitting. The standard deviation of the second responses conditional on the fixed and random effects was moderate (sigma = .12, 95% CI [.12, .12]).

## Supplementary Table 3

Results from Bayesian model estimating susceptibility to **prosocial and anti-social influence**, at **time 1**

| **BRMS Output**  Family: gaussian  Links: mu = identity; sigma = identity  Formula: rating2_recoded ~ 1 + rating1_recoded + delta_positive + delta_negative + (0 + delta_positive + delta_negative \| scenario) + (0 + delta_positive + delta_negative \| id)  Data: dat_time1 (Number of observations: 7200)  Draws: 7 chains, each with iter = 4000; warmup = 1500; thin = 1; post-warmup draws = 17500 | | | | | | | | |
| --- | --- | --- | --- | --- | --- | --- | --- | --- |
| **Variable** | **Estimate** | **Est. Error** | **95% CI LB** | **95% CI UB** | **prior** | **Rhat** | **Bulk ESS** | **Tail ESS** |
| **Group-Level (Random) Effects:** | | | | | | | | |
| **Participants (Number of Levels: 528)** | | | | | | | | |
| SD(delta^+^) | .25 | .01 | .23 | .28 | student_t(7, 0, .75) | 1.00 | 5872 | 6858 |
| SD(delta^−^) | .12 | .01 | .11 | .14 | student_t(7, 0, .75) | 1.00 | 7245 | 10965 |
| cor(delta^+^, delta−) | .39 | .06 | .25 | .51 | lkj_corr_cholesky(2.5) | 1.00 | 4061 | 8261 |
| **Scenario (Number of Levels: 82)** | | | | | | | | |
| SD(delta^+^) | .09 | .01 | .06 | .12 | student_t(7, 0, .75) | 1.00 | 5764 | 9402 |
| SD(delta^−^) | .05 | .01 | .03 | .06 | student_t(7, 0, .75) | 1.00 | 7226 | 11039 |
| cor(delta^+^, delta−) | -.42 | .16 | -.72 | -.08 | lkj_corr_cholesky(2.5) | 1.00 | 5360 | 8689 |
| **Population-Level (Fixed) Effects:** | | | | | | | | |
| Intercept | .02 | .01 | .01 | .03 | student_t(3, .7, 2.5) | 1.00 | 22647 | 13963 |
| Initial Rating | .98 | .01 | .96 | .99 | student_t(7, 0, .75) | 1.00 | 23847 | 13203 |
| delta^+^ | .26 | .02 | .22 | .30 | student_t(7, 0, .75) | 1.00 | 7034 | 9703 |
| delta^−^ | .12 | .01 | .10 | .14 | student_t(7, 0, .75) | 1.00 | 10515 | 12299 |
| **Family Specific Parameters** | | | | | | | | |
| sigma | .12 | .00 | .12 | .12 | student_t(3, 0, 2.5) | 1.00 | 16738 | 12585 |

Note. student_t(df, mu, sigma) refers to a student-T distribution prior with a degrees of freedom df, location mu, and scale sigma. lkj_corr_cholesky(eta) refers to a LKJ distribution prior for the lower-triangular Cholesky factor of a correlation matrix with shape eta.

## Supplementary Table 4

Results from Bayesian model estimating susceptibility to **prosocial and anti-social influence**, at **time 2**

| **BRMS Output**  Family: gaussian  Links: mu = identity; sigma = identity  Formula: rating2_recoded ~ 1 + rating1_recoded + delta_positive + delta_negative + (0 + delta_positive + delta_negative \| scenario) + (0 + delta_positive + delta_negative \| id)  Data: dat_time2 (Number of observations: 5856)  Draws: 7 chains, each with iter = 4000; warmup = 1500; thin = 1;  total post-warmup draws = 17500 | | | | | | | | |
| --- | --- | --- | --- | --- | --- | --- | --- | --- |
| **Variable** | **Estimate** | **Est. Error** | **95% CI LB** | **95% CI UB** | **prior** | **Rhat** | **Bulk ESS** | **Tail ESS** |
| **Group-Level (Random) Effects:** | | | | | | | | |
| **Participants (Number of Levels: 528)** | | | | | | | | |
| SD(delta^+^) | .25 | .02 | .22 | .28 | student_t(7, 0, .75) | 1.00 | 5373 | 9281 |
| SD(delta^−^) | .10 | .01 | .08 | .11 | student_t(7, 0, .75) | 1.00 | 6846 | 9779 |
| cor(delta^+^, delta−) | .35 | .08 | .19 | .49 | lkj_corr_cholesky(2.5) | 1.00 | 5692 | 9561 |
| **Scenario (Number of Levels: 82)** | | | | | | | | |
| SD(delta^+^) | .09 | .01 | .06 | .12 | student_t(7, 0, .75) | 1.00 | 6109 | 10222 |
| SD(delta^−^) | .02 | .01 | .01 | .04 | student_t(7, 0, .75) | 1.00 | 3957 | 3286 |
| cor(delta^+^, delta−) | -.31 | .27 | -.78 | .25 | lkj_corr_cholesky(2.5) | 1.00 | 8437 | 9866 |
| **Population-Level (Fixed) Effects:** | | | | | | | | |
| Intercept | .02 | .01 | .01 | .03 | student_t(3, .7, 2.5) | 1.00 | 23000 | 15103 |
| Initial Rating | .98 | .01 | .96 | 1.00 | student_t(7, 0, .75) | 1.00 | 22668 | 13485 |
| delta^+^ | .23 | .02 | .18 | .27 | student_t(7, 0, .75) | 1.00 | 7275 | 10440 |
| delta^−^ | .09 | .01 | .07 | .11 | student_t(7, 0, .75) | 1.00 | 14585 | 14057 |
| **Family Specific Parameters** | | | | | | | | |
| sigma | .12 | .00 | .11 | .12 | student_t(3, 0, 2.5) | 1.00 | 17864 | 12381 |

Note. student_t(df, mu, sigma) refers to a student-T distribution prior with a degrees of freedom df, location mu, and scale sigma. lkj_corr_cholesky(eta) refers to a LKJ distribution prior for the lower-triangular Cholesky factor of a correlation matrix with shape eta.

## Supplementary Figure 1

Distribution of response dates to the time 4 COVID questionnaire. Changes in the national COVID regulations are highlighted across this period.


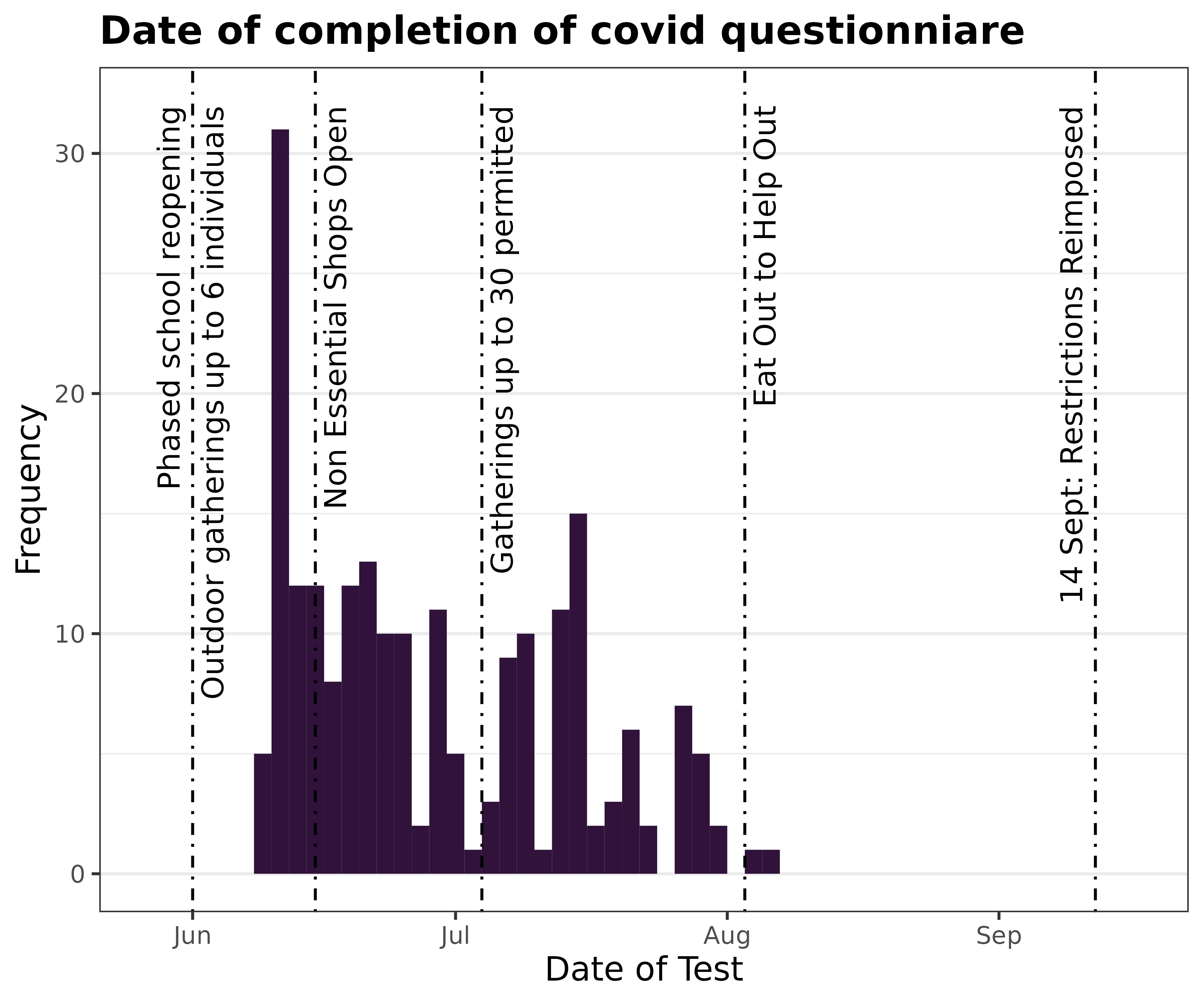


## Supplementary Figure 2

Distribution of missing data across variables.


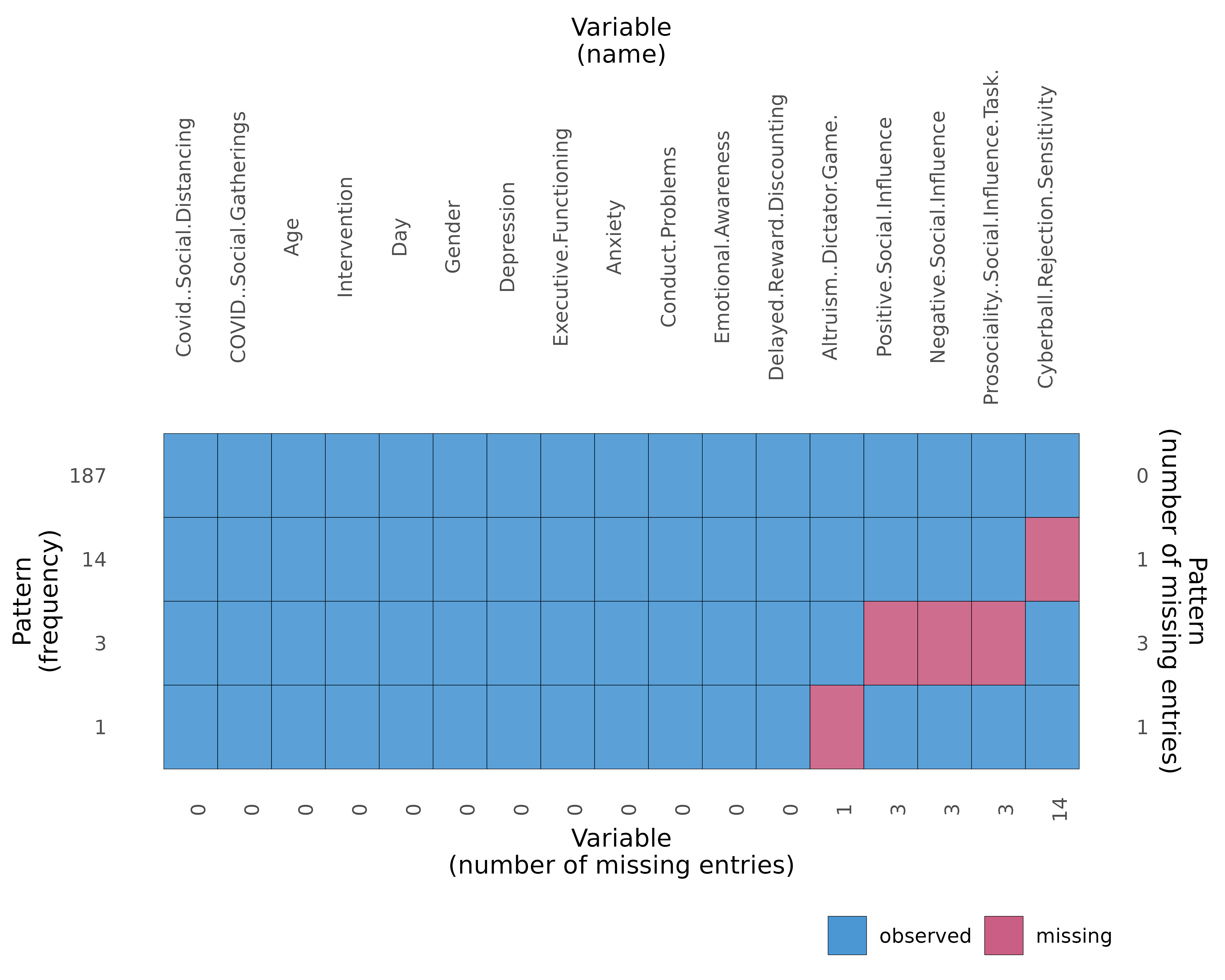


*Note.* This plot is generated by the R function *mice::plot_pattern*. The plot indicates how many participants have specific patterns of missing data. For example, the top row indicates that 187 individuals have no missing data, and the second row tells us that 14 participants have complete data except for missing rejection sensitivity,

## Supplementary Figure 3

Correlations between all variables are shown on the lower diagonal. The number of non-missing responses for each variable is shown on the diagonal. The sample size for each correlation (i.e. the number of participants with data on both variables) and the 95% confidence interval is shown on the upper diagonal. Pre-pandemic variables, except for rejection sensitivity, have been averaged across Time 1 & 2.


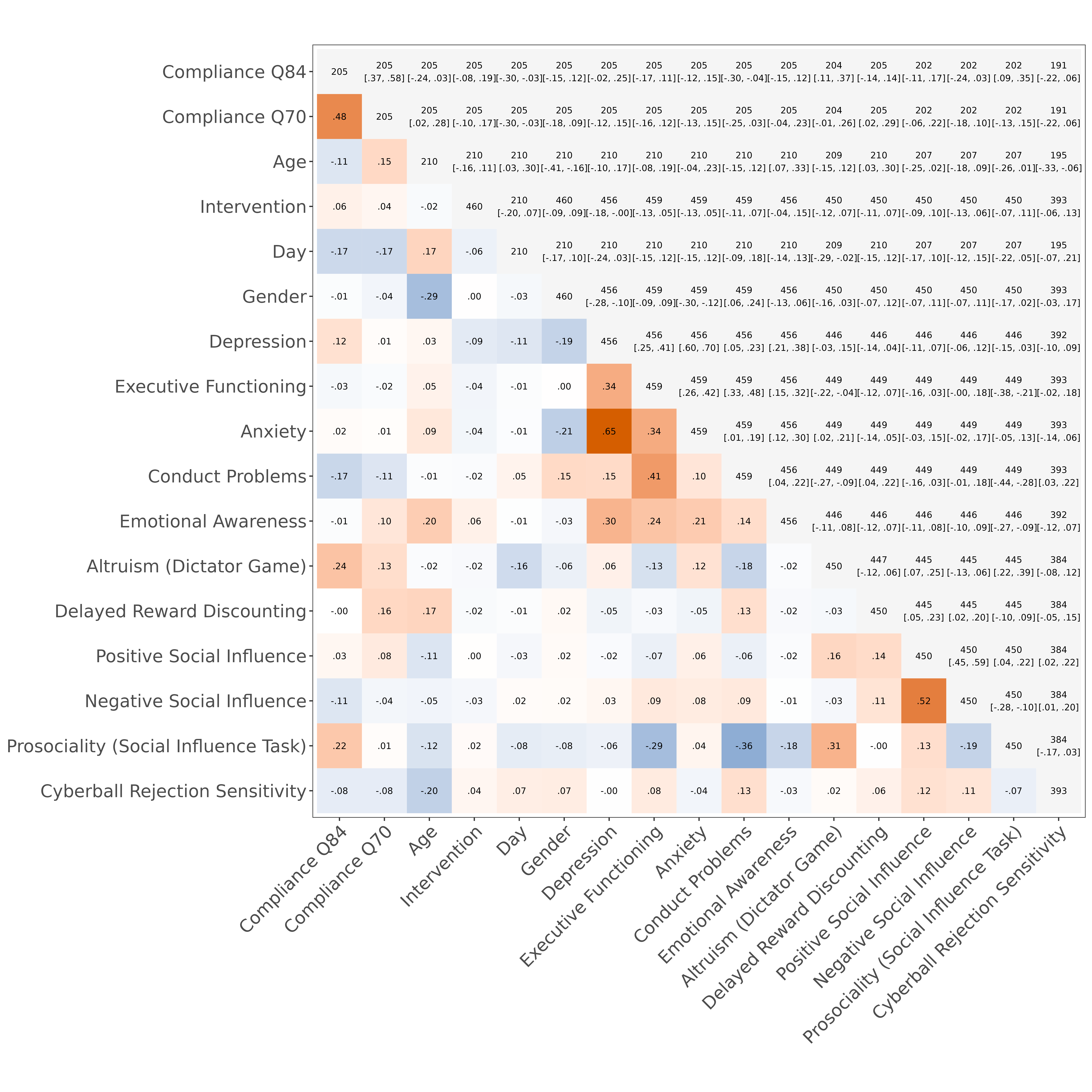


## Supplementary Figure 4

Factor loadings from the five-factor model of well-being, behaviour and mental health variables


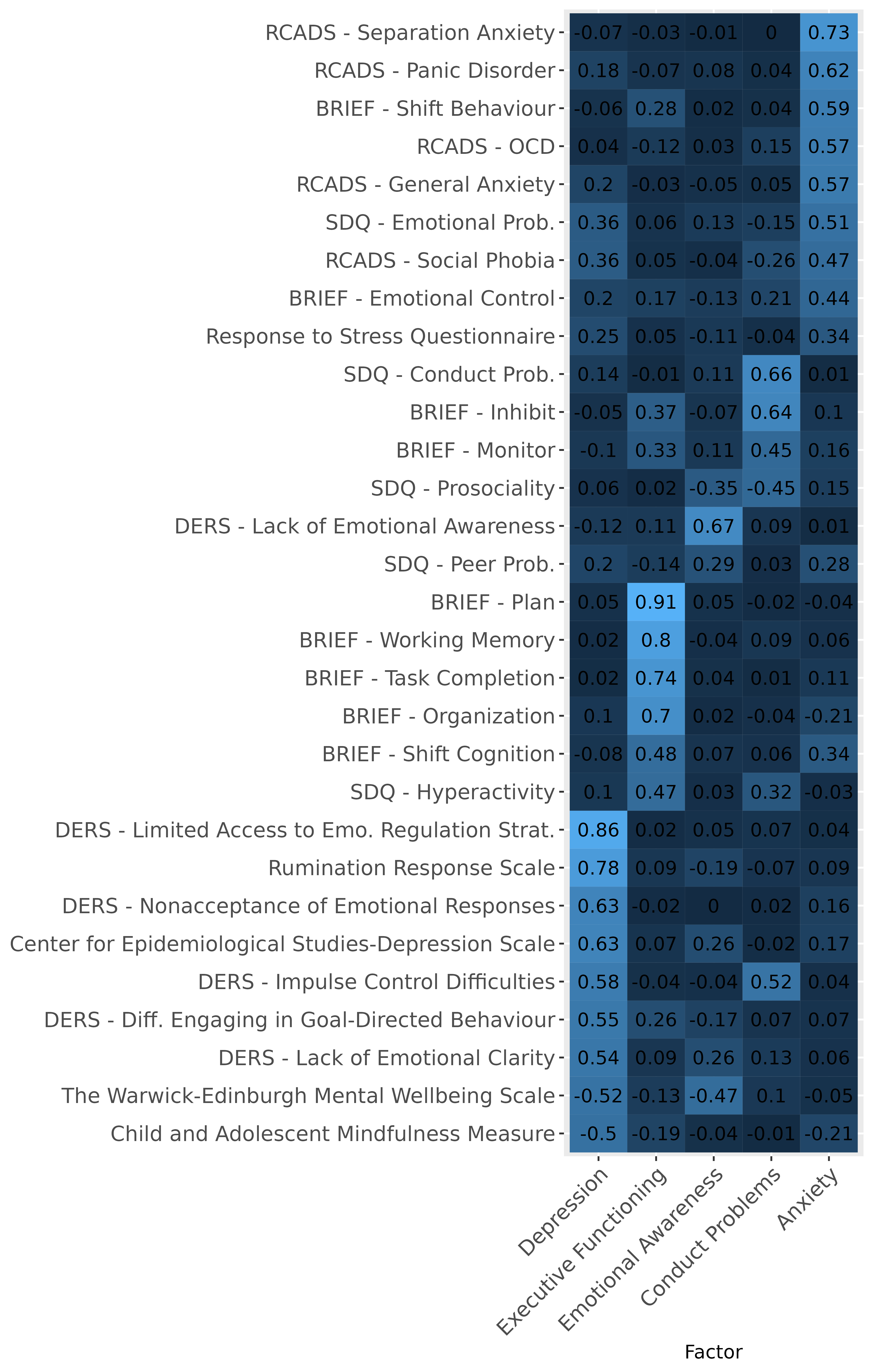


## Model Results for Primary Outcome

As described in Statistical Analysis, we run separate models for each predictor controlling for the four covariates. Each separate model is reported below, along with the covariate effects and intercepts not reported in the manuscript. The ordinal regression models estimate four intercept terms because the ordinal outcome measures have 5 levels. Rhat, Bulk ESS and Tail ESS measure the quality of MCMC sampling. Rhat is a convergence diagnostic measure; Bulk ESS measures the efficiency of estimating the posterior distribution, and tail ESS measures the efficiency in estimating the tail of the posterior distribution (and hence the credible intervals). N is the sample size of each model after excluding missing observations.

| Variable | Estimate | Est.Error | l-95% CI | u-95% CI | Rhat | Bulk_ESS | Tail_ESS | N |
| --- | --- | --- | --- | --- | --- | --- | --- | --- |
| **Depression** | | | | | | | | |
| Intercept[1] | -2.045 | .207 | -2.462 | -1.657 | 1.00 | 20161 | 18352 | 205 |
| Intercept[2] | -1.399 | .155 | -1.705 | -1.099 | 1.00 | 31491 | 23918 | 205 |
| Intercept[3] | -.818 | .138 | -1.089 | -.553 | 1.00 | 31582 | 24706 | 205 |
| Intercept[4] | .550 | .135 | .285 | .816 | 1.00 | 30489 | 24915 | 205 |
| Age | -.107 | .082 | -.265 | .054 | 1.00 | 29913 | 21632 | 205 |
| Intervention | .100 | .154 | -.200 | .402 | 1.00 | 35065 | 20023 | 205 |
| Day | -.171 | .079 | -.326 | -.015 | 1.00 | 31562 | 20730 | 205 |
| Gender | -.041 | .170 | -.373 | .294 | 1.00 | 31439 | 22132 | 205 |
| Depression | .127 | .084 | -.040 | .292 | 1.00 | 31325 | 21473 | 205 |
| **Executive Functioning** | | | | | | | | |
| Intercept[1] | -2.036 | .206 | -2.453 | -1.647 | 1.00 | 20870 | 19490 | 205 |
| Intercept[2] | -1.405 | .156 | -1.715 | -1.104 | 1.00 | 30469 | 21927 | 205 |
| Intercept[3] | -.834 | .139 | -1.110 | -.561 | 1.00 | 29417 | 23257 | 205 |
| Intercept[4] | .531 | .135 | .267 | .799 | 1.00 | 29339 | 23537 | 205 |
| Age | -.096 | .081 | -.254 | .063 | 1.00 | 30695 | 21080 | 205 |
| Intervention | .084 | .153 | -.214 | .386 | 1.00 | 32777 | 20955 | 205 |
| Day | -.178 | .079 | -.334 | -.024 | 1.00 | 31574 | 21399 | 205 |
| Gender | -.076 | .168 | -.404 | .252 | 1.00 | 29931 | 21383 | 205 |
| Executive Functioning | -.033 | .081 | -.193 | .129 | 1.00 | 36823 | 21213 | 205 |
| **Anxiety** | | | | | | | | |
| Intercept[1] | -2.035 | .205 | -2.456 | -1.652 | 1.00 | 20277 | 19012 | 205 |
| Intercept[2] | -1.404 | .155 | -1.713 | -1.102 | 1.00 | 29395 | 22423 | 205 |
| Intercept[3] | -.831 | .138 | -1.105 | -.560 | 1.00 | 29298 | 23205 | 205 |
| Intercept[4] | .533 | .135 | .268 | .799 | 1.00 | 29449 | 23184 | 205 |
| Age | -.100 | .081 | -.261 | .060 | 1.00 | 31082 | 21325 | 205 |
| Intervention | .090 | .154 | -.211 | .390 | 1.00 | 32043 | 20791 | 205 |
| Day | -.179 | .079 | -.335 | -.024 | 1.00 | 31766 | 21278 | 205 |
| Gender | -.076 | .169 | -.408 | .255 | 1.00 | 28823 | 21226 | 205 |
| Anxiety | .009 | .078 | -.143 | .165 | 1.00 | 30693 | 21571 | 205 |
| **Conduct Problems** | | | | | | | | |
| Intercept[1] | -2.002 | .208 | -2.426 | -1.610 | 1.00 | 20883 | 17567 | 205 |
| Intercept[2] | -1.363 | .157 | -1.673 | -1.059 | 1.00 | 28717 | 21556 | 205 |
| Intercept[3] | -.784 | .140 | -1.062 | -.515 | 1.00 | 28315 | 23539 | 205 |
| Intercept[4] | .600 | .138 | .328 | .871 | 1.00 | 26712 | 23452 | 205 |
| Age | -.097 | .081 | -.255 | .063 | 1.00 | 28442 | 20799 | 205 |
| Intervention | .101 | .154 | -.197 | .403 | 1.00 | 31843 | 21168 | 205 |
| Day | -.165 | .079 | -.320 | -.010 | 1.00 | 30890 | 20925 | 205 |
| Gender | .019 | .173 | -.318 | .357 | 1.00 | 28154 | 21571 | 205 |
| Conduct Problems | -.184 | .086 | -.353 | -.013 | 1.00 | 27943 | 20904 | 205 |
| **Emotional Awareness** | | | | | | | | |
| Intercept[1] | -2.038 | .205 | -2.452 | -1.651 | 1.00 | 21276 | 19850 | 205 |
| Intercept[2] | -1.406 | .155 | -1.718 | -1.105 | 1.00 | 32365 | 21444 | 205 |
| Intercept[3] | -.835 | .138 | -1.107 | -.566 | 1.00 | 32527 | 24158 | 205 |
| Intercept[4] | .529 | .134 | .265 | .789 | 1.00 | 31761 | 24324 | 205 |
| Age | -.090 | .086 | -.258 | .078 | 1.00 | 27688 | 20991 | 205 |
| Intervention | .088 | .153 | -.213 | .393 | 1.00 | 36952 | 19744 | 205 |
| Day | -.180 | .079 | -.333 | -.025 | 1.00 | 33129 | 20666 | 205 |
| Gender | -.076 | .168 | -.406 | .253 | 1.00 | 30661 | 21708 | 205 |
| Emotional Awareness | -.029 | .080 | -.185 | .129 | 1.00 | 30417 | 20992 | 205 |
| **Altruism (Dictator Game)** | | | | | | | | |
| Intercept[1] | -2.081 | .210 | -2.503 | -1.683 | 1.00 | 19694 | 17611 | 204 |
| Intercept[2] | -1.427 | .158 | -1.738 | -1.120 | 1.00 | 29959 | 24026 | 204 |
| Intercept[3] | -.839 | .139 | -1.110 | -.569 | 1.00 | 29173 | 22218 | 204 |
| Intercept[4] | .556 | .136 | .291 | .821 | 1.00 | 27927 | 23539 | 204 |
| Age | -.124 | .082 | -.285 | .037 | 1.00 | 28349 | 21607 | 204 |
| Intervention | .088 | .155 | -.214 | .394 | 1.00 | 31291 | 20631 | 204 |
| Day | -.141 | .080 | -.298 | .016 | 1.00 | 29041 | 21831 | 204 |
| Gender | -.081 | .167 | -.408 | .243 | 1.00 | 28834 | 20746 | 204 |
| Altruism (Dictator Game) | .270 | .081 | .111 | .427 | 1.00 | 29195 | 21387 | 204 |
| **Delayed Reward Discounting** | | | | | | | | |
| Intercept[1] | -2.036 | .205 | -2.447 | -1.647 | 1.00 | 22016 | 20611 | 205 |
| Intercept[2] | -1.402 | .157 | -1.712 | -1.098 | 1.00 | 31179 | 23309 | 205 |
| Intercept[3] | -.829 | .140 | -1.102 | -.556 | 1.00 | 30651 | 23954 | 205 |
| Intercept[4] | .536 | .136 | .272 | .803 | 1.00 | 30189 | 23405 | 205 |
| Age | -.093 | .083 | -.255 | .070 | 1.00 | 29814 | 21748 | 205 |
| Intervention | .081 | .153 | -.218 | .382 | 1.00 | 31601 | 20130 | 205 |
| Day | -.180 | .080 | -.337 | -.022 | 1.00 | 31271 | 20077 | 205 |
| Gender | -.071 | .169 | -.402 | .259 | 1.00 | 30257 | 21497 | 205 |
| Delayed Reward Discounting | -.039 | .099 | -.232 | .154 | 1.00 | 30942 | 21326 | 205 |
| **Prosocial Influence** | | | | | | | | |
| Intercept[1] | -2.034 | .205 | -2.453 | -1.645 | 1.00 | 21992 | 20571 | 205 |
| Intercept[2] | -1.404 | .155 | -1.713 | -1.104 | 1.00 | 32686 | 22874 | 205 |
| Intercept[3] | -.834 | .138 | -1.104 | -.563 | 1.00 | 32583 | 22559 | 205 |
| Intercept[4] | .530 | .134 | .267 | .792 | 1.00 | 31528 | 24161 | 205 |
| Age | -.100 | .083 | -.263 | .063 | 1.00 | 30635 | 21852 | 205 |
| Intervention | .086 | .151 | -.211 | .381 | 1.00 | 34691 | 20412 | 205 |
| Day | -.178 | .079 | -.332 | -.024 | 1.00 | 32799 | 21322 | 205 |
| Gender | -.077 | .167 | -.405 | .251 | 1.00 | 30705 | 21688 | 205 |
| Prosocial Influence | -.010 | .087 | -.182 | .161 | 1.00 | 32055 | 22002 | 205 |
| **Anti-social Influence** | | | | | | | | |
| Intercept[1] | -2.039 | .206 | -2.453 | -1.650 | 1.00 | 20538 | 20275 | 205 |
| Intercept[2] | -1.406 | .154 | -1.712 | -1.109 | 1.00 | 32359 | 24148 | 205 |
| Intercept[3] | -.830 | .138 | -1.103 | -.566 | 1.00 | 30959 | 23010 | 205 |
| Intercept[4] | .571 | .137 | .305 | .843 | 1.00 | 30966 | 24275 | 205 |
| Age | -.113 | .083 | -.275 | .048 | 1.00 | 30329 | 21116 | 205 |
| Intervention | .059 | .154 | -.240 | .362 | 1.00 | 34182 | 21490 | 205 |
| Day | -.185 | .080 | -.342 | -.029 | 1.00 | 30223 | 20351 | 205 |
| Gender | -.053 | .168 | -.383 | .277 | 1.00 | 32993 | 21496 | 205 |
| Anti-social Influence | -.271 | .094 | -.455 | -.087 | 1.00 | 32421 | 21115 | 205 |
| **Prosociality (Social Influence Task)** | | | | | | | | |
| Intercept[1] | -2.045 | .203 | -2.454 | -1.658 | 1.00 | 22742 | 19821 | 205 |
| Intercept[2] | -1.426 | .155 | -1.732 | -1.125 | 1.00 | 32524 | 23144 | 205 |
| Intercept[3] | -.850 | .138 | -1.120 | -.578 | 1.00 | 32585 | 22877 | 205 |
| Intercept[4] | .559 | .135 | .295 | .824 | 1.00 | 32027 | 24249 | 205 |
| Age | -.058 | .082 | -.220 | .104 | 1.00 | 31622 | 21525 | 205 |
| Intervention | .058 | .154 | -.242 | .357 | 1.00 | 34874 | 20884 | 205 |
| Day | -.161 | .080 | -.319 | -.004 | 1.00 | 35294 | 21838 | 205 |
| Gender | -.001 | .169 | -.333 | .332 | 1.00 | 31268 | 20693 | 205 |
| Prosociality (Social Influence Task) | .262 | .081 | .103 | .423 | 1.00 | 34335 | 21463 | 205 |
| **Cyberball Rejection Sensitivity** | | | | | | | | |
| Intercept[1] | -2.164 | .229 | -2.633 | -1.736 | 1.00 | 20003 | 19498 | 191 |
| Intercept[2] | -1.441 | .163 | -1.766 | -1.122 | 1.00 | 30369 | 22717 | 191 |
| Intercept[3] | -.835 | .143 | -1.114 | -.554 | 1.00 | 30413 | 23907 | 191 |
| Intercept[4] | .518 | .140 | .247 | .794 | 1.00 | 31342 | 23516 | 191 |
| Age | -.098 | .087 | -.269 | .073 | 1.00 | 28080 | 21710 | 191 |
| Intervention | .048 | .158 | -.264 | .355 | 1.00 | 32652 | 21406 | 191 |
| Day | -.153 | .082 | -.315 | .009 | 1.00 | 31488 | 20746 | 191 |
| Gender | -.015 | .171 | -.349 | .324 | 1.00 | 32014 | 21986 | 191 |
| Cyberball Rejection Sensitivity | -.125 | .085 | -.293 | .042 | 1.00 | 29969 | 20883 | 191 |

## Model Results for Secondary Outcome

| Variable | Estimate | Est.Error | l-95% CI | u-95% CI | Rhat | Bulk_ESS | Tail_ESS | N |
| --- | --- | --- | --- | --- | --- | --- | --- | --- |
| **Depression** | | | | | | | | |
| Intercept[1] | -2.864 | .345 | -3.592 | -2.243 | 1.00 | 19911 | 19326 | 205 |
| Intercept[2] | -2.197 | .227 | -2.659 | -1.769 | 1.00 | 35792 | 21863 | 205 |
| Intercept[3] | -1.828 | .189 | -2.204 | -1.460 | 1.00 | 32510 | 23157 | 205 |
| Intercept[4] | -1.151 | .156 | -1.461 | -.850 | 1.00 | 29280 | 22699 | 205 |
| Intercept[5] | -.160 | .141 | -.437 | .116 | 1.00 | 30623 | 24081 | 205 |
| Age | .186 | .087 | .016 | .357 | 1.00 | 30926 | 21423 | 205 |
| Intervention | .052 | .168 | -.276 | .379 | 1.00 | 30456 | 18540 | 205 |
| Day | -.234 | .084 | -.400 | -.069 | 1.00 | 32276 | 21478 | 205 |
| Gender | .027 | .180 | -.325 | .381 | 1.00 | 32182 | 21834 | 205 |
| Depression | .003 | .089 | -.170 | .176 | 1.00 | 31601 | 21357 | 205 |
| **Executive Functioning** | | | | | | | | |
| Intercept[1] | -2.865 | .351 | -3.609 | -2.230 | 1.00 | 19427 | 18135 | 205 |
| Intercept[2] | -2.198 | .229 | -2.665 | -1.771 | 1.00 | 34938 | 21556 | 205 |
| Intercept[3] | -1.829 | .190 | -2.212 | -1.468 | 1.00 | 34606 | 21426 | 205 |
| Intercept[4] | -1.152 | .155 | -1.459 | -.853 | 1.00 | 31330 | 23605 | 205 |
| Intercept[5] | -.161 | .140 | -.438 | .111 | 1.00 | 31068 | 24411 | 205 |
| Age | .187 | .087 | .018 | .358 | 1.00 | 29121 | 21328 | 205 |
| Intervention | .052 | .168 | -.277 | .380 | 1.00 | 33492 | 20080 | 205 |
| Day | -.234 | .085 | -.402 | -.067 | 1.00 | 34834 | 20617 | 205 |
| Gender | .026 | .180 | -.330 | .380 | 1.00 | 31329 | 21403 | 205 |
| Executive Functioning | -.002 | .087 | -.173 | .168 | 1.00 | 33622 | 20378 | 205 |
| **Anxiety** | | | | | | | | |
| Intercept[1] | -2.877 | .350 | -3.618 | -2.247 | 1.00 | 19967 | 17378 | 205 |
| Intercept[2] | -2.204 | .227 | -2.662 | -1.773 | 1.00 | 32844 | 20605 | 205 |
| Intercept[3] | -1.835 | .189 | -2.216 | -1.474 | 1.00 | 31847 | 21251 | 205 |
| Intercept[4] | -1.156 | .155 | -1.463 | -.852 | 1.00 | 29073 | 22790 | 205 |
| Intercept[5] | -.167 | .140 | -.443 | .105 | 1.00 | 28709 | 23149 | 205 |
| Age | .187 | .088 | .015 | .358 | 1.00 | 30178 | 21861 | 205 |
| Intervention | .048 | .165 | -.278 | .369 | 1.00 | 31705 | 20667 | 205 |
| Day | -.234 | .084 | -.397 | -.067 | 1.00 | 32268 | 21170 | 205 |
| Gender | .017 | .182 | -.340 | .371 | 1.00 | 30116 | 20707 | 205 |
| Anxiety | -.019 | .084 | -.183 | .147 | 1.00 | 30267 | 20486 | 205 |
| **Conduct Problems** | | | | | | | | |
| Intercept[1] | -2.848 | .348 | -3.575 | -2.216 | 1.00 | 19361 | 17796 | 205 |
| Intercept[2] | -2.174 | .230 | -2.636 | -1.741 | 1.00 | 33832 | 22599 | 205 |
| Intercept[3] | -1.803 | .191 | -2.185 | -1.438 | 1.00 | 33466 | 23331 | 205 |
| Intercept[4] | -1.123 | .156 | -1.431 | -.818 | 1.00 | 29081 | 23624 | 205 |
| Intercept[5] | -.129 | .142 | -.407 | .146 | 1.00 | 28262 | 23333 | 205 |
| Age | .191 | .087 | .021 | .362 | 1.00 | 29485 | 21276 | 205 |
| Intervention | .058 | .164 | -.261 | .381 | 1.00 | 31676 | 20521 | 205 |
| Day | -.229 | .085 | -.395 | -.063 | 1.00 | 32204 | 20527 | 205 |
| Gender | .081 | .187 | -.288 | .450 | 1.00 | 28448 | 20664 | 205 |
| Conduct Problems | -.103 | .092 | -.282 | .079 | 1.00 | 31823 | 21583 | 205 |
| **Emotional Awareness** | | | | | | | | |
| Intercept[1] | -2.873 | .347 | -3.600 | -2.253 | 1.00 | 16587 | 17882 | 205 |
| Intercept[2] | -2.203 | .226 | -2.666 | -1.774 | 1.00 | 33731 | 22336 | 205 |
| Intercept[3] | -1.833 | .189 | -2.213 | -1.469 | 1.00 | 32613 | 23563 | 205 |
| Intercept[4] | -1.154 | .154 | -1.458 | -.855 | 1.00 | 28789 | 22724 | 205 |
| Intercept[5] | -.158 | .140 | -.433 | .111 | 1.00 | 28587 | 23603 | 205 |
| Age | .161 | .091 | -.016 | .340 | 1.00 | 24260 | 20880 | 205 |
| Intervention | .050 | .164 | -.275 | .372 | 1.00 | 30767 | 21772 | 205 |
| Day | -.230 | .084 | -.394 | -.066 | 1.00 | 30102 | 20440 | 205 |
| Gender | .021 | .181 | -.337 | .376 | 1.00 | 29627 | 21073 | 205 |
| Emotional Awareness | .086 | .087 | -.084 | .257 | 1.00 | 28243 | 21007 | 205 |
| **Altruism (Dictator Game)** | | | | | | | | |
| Intercept[1] | -2.860 | .348 | -3.598 | -2.239 | 1.00 | 18987 | 18507 | 204 |
| Intercept[2] | -2.196 | .226 | -2.652 | -1.771 | 1.00 | 31709 | 22713 | 204 |
| Intercept[3] | -1.834 | .190 | -2.211 | -1.470 | 1.00 | 32205 | 22695 | 204 |
| Intercept[4] | -1.167 | .155 | -1.474 | -.869 | 1.00 | 27854 | 23155 | 204 |
| Intercept[5] | -.174 | .141 | -.452 | .101 | 1.00 | 28246 | 23239 | 204 |
| Age | .168 | .088 | -.007 | .339 | 1.00 | 27216 | 20377 | 204 |
| Intervention | .047 | .166 | -.280 | .374 | 1.00 | 28562 | 19949 | 204 |
| Day | -.206 | .087 | -.377 | -.036 | 1.00 | 30364 | 21750 | 204 |
| Gender | .019 | .179 | -.330 | .370 | 1.00 | 29287 | 21445 | 204 |
| Altruism (Dictator Game) | .208 | .087 | .039 | .376 | 1.00 | 31121 | 21293 | 204 |
| **Delayed Reward Discounting** | | | | | | | | |
| Intercept[1] | -2.901 | .349 | -3.651 | -2.276 | 1.00 | 20673 | 17529 | 205 |
| Intercept[2] | -2.229 | .232 | -2.702 | -1.794 | 1.00 | 35928 | 22066 | 205 |
| Intercept[3] | -1.858 | .193 | -2.244 | -1.490 | 1.00 | 33595 | 21980 | 205 |
| Intercept[4] | -1.182 | .157 | -1.492 | -.879 | 1.00 | 30375 | 22796 | 205 |
| Intercept[5] | -.190 | .142 | -.472 | .087 | 1.00 | 29916 | 23345 | 205 |
| Age | .169 | .088 | -.003 | .342 | 1.00 | 29827 | 21530 | 205 |
| Intervention | .067 | .166 | -.258 | .391 | 1.00 | 33635 | 20160 | 205 |
| Day | -.233 | .085 | -.400 | -.066 | 1.00 | 31085 | 19229 | 205 |
| Gender | -.005 | .182 | -.365 | .355 | 1.00 | 31405 | 19706 | 205 |
| Delayed Reward Discounting | .151 | .114 | -.070 | .374 | 1.00 | 33278 | 20396 | 205 |
| **Prosocial Influence** | | | | | | | | |
| Intercept[1] | -2.883 | .348 | -3.621 | -2.260 | 1.00 | 16670 | 16986 | 205 |
| Intercept[2] | -2.211 | .227 | -2.675 | -1.783 | 1.00 | 30632 | 20283 | 205 |
| Intercept[3] | -1.843 | .189 | -2.217 | -1.479 | 1.00 | 29165 | 23413 | 205 |
| Intercept[4] | -1.166 | .154 | -1.468 | -.870 | 1.00 | 28508 | 23573 | 205 |
| Intercept[5] | -.171 | .139 | -.442 | .103 | 1.00 | 28467 | 23667 | 205 |
| Age | .209 | .089 | .037 | .384 | 1.00 | 26087 | 20309 | 205 |
| Intervention | .056 | .165 | -.268 | .378 | 1.00 | 29023 | 20872 | 205 |
| Day | -.236 | .085 | -.403 | -.067 | 1.00 | 28102 | 21311 | 205 |
| Gender | .016 | .180 | -.339 | .369 | 1.00 | 28723 | 21686 | 205 |
| Prosocial Influence | .126 | .099 | -.065 | .323 | 1.00 | 29527 | 21768 | 205 |
| **Anti-social Influence** | | | | | | | | |
| Intercept[1] | -2.863 | .347 | -3.596 | -2.238 | 1.00 | 19080 | 18367 | 205 |
| Intercept[2] | -2.196 | .227 | -2.666 | -1.770 | 1.00 | 35325 | 22031 | 205 |
| Intercept[3] | -1.825 | .190 | -2.207 | -1.462 | 1.00 | 33247 | 22856 | 205 |
| Intercept[4] | -1.147 | .155 | -1.455 | -.844 | 1.00 | 31070 | 22478 | 205 |
| Intercept[5] | -.155 | .140 | -.430 | .115 | 1.00 | 31126 | 22702 | 205 |
| Age | .185 | .088 | .012 | .357 | 1.00 | 31096 | 21077 | 205 |
| Intervention | .043 | .167 | -.286 | .369 | 1.00 | 35082 | 20312 | 205 |
| Day | -.237 | .085 | -.402 | -.070 | 1.00 | 33618 | 20821 | 205 |
| Gender | .036 | .181 | -.317 | .391 | 1.00 | 32450 | 20889 | 205 |
| Anti-social Influence | -.065 | .100 | -.258 | .131 | 1.00 | 33387 | 19551 | 205 |
| **Prosociality (Social Influence Task)** | | | | | | | | |
| Intercept[1] | -2.866 | .347 | -3.603 | -2.244 | 1.00 | 16982 | 17210 | 205 |
| Intercept[2] | -2.201 | .225 | -2.659 | -1.775 | 1.00 | 32244 | 22676 | 205 |
| Intercept[3] | -1.831 | .188 | -2.207 | -1.469 | 1.00 | 30368 | 23255 | 205 |
| Intercept[4] | -1.153 | .154 | -1.452 | -.855 | 1.00 | 29335 | 23006 | 205 |
| Intercept[5] | -.162 | .140 | -.438 | .110 | 1.00 | 29274 | 23208 | 205 |
| Age | .186 | .088 | .013 | .358 | 1.00 | 26477 | 21377 | 205 |
| Intervention | .051 | .166 | -.272 | .377 | 1.00 | 30288 | 21355 | 205 |
| Day | -.234 | .084 | -.400 | -.068 | 1.00 | 29067 | 21019 | 205 |
| Gender | .023 | .182 | -.332 | .380 | 1.00 | 26596 | 20979 | 205 |
| Prosociality (Social Influence Task) | .002 | .084 | -.162 | .169 | 1.00 | 28485 | 20071 | 205 |
| **Cyberball Rejection Sensitivity** | | | | | | | | |
| Intercept[1] | -2.859 | .354 | -3.604 | -2.226 | 1.00 | 18360 | 17608 | 191 |
| Intercept[2] | -2.181 | .233 | -2.652 | -1.742 | 1.00 | 34396 | 22048 | 191 |
| Intercept[3] | -1.806 | .195 | -2.195 | -1.426 | 1.00 | 31866 | 22660 | 191 |
| Intercept[4] | -1.218 | .164 | -1.543 | -.897 | 1.00 | 27775 | 22695 | 191 |
| Intercept[5] | -.176 | .146 | -.463 | .108 | 1.00 | 30296 | 23878 | 191 |
| Age | .181 | .094 | -.003 | .364 | 1.00 | 27601 | 21892 | 191 |
| Intervention | .009 | .171 | -.329 | .346 | 1.00 | 32403 | 21093 | 191 |
| Day | -.264 | .088 | -.437 | -.091 | 1.00 | 32721 | 20217 | 191 |
| Gender | .051 | .185 | -.310 | .414 | 1.00 | 31279 | 20290 | 191 |
| Cyberball Rejection Sensitivity | -.032 | .090 | -.208 | .147 | 1.00 | 31180 | 21526 | 191 |

1. Note that CI = credible interval in this section [↑](#footnote-ref-2)
